# Allelic Variation in *Zmfatb* Gene Defines Variability for Fatty Acids Composition Among Diverse Maize Genotypes

**DOI:** 10.3389/fnut.2022.845255

**Published:** 2022-05-06

**Authors:** Ashvinkumar Katral, Vignesh Muthusamy, Rajkumar U. Zunjare, Rashmi Chhabra, Shalma Maman, Devendra K. Yadava, Firoz Hossain

**Affiliations:** Division of Genetics, ICAR-Indian Agricultural Research Institute, New Delhi, India

**Keywords:** maize, fatty acids, acyl-ACP thioesterase, allelic variation, haplotypes, molecular docking, PUFA, corn oil

## Abstract

Edible oil with lower saturated fatty acids is desired for perceived quality and health benefits to humans and livestock. *fatb* gene encoding acyl-ACP thioesterase is a key player in the conversion of palmitic acid to oleic acid, thereby modifying the ratio of saturated to unsaturated fatty acids in maize kernels. The present investigation characterised the full-length sequence of the *Zmfatb* gene (4.63 kb) in two mutants (*Zmfatb*) and eight wild-types (*ZmfatB*) inbreds to study allelic variation, gene-based diversity, phylogenetic-relationship, protein-modelling, and molecular-docking to identify novel candidates for modification of fatty acid profile. Sequence alignment revealed wide genomic variability for *Zmfatb* among the inbreds; identified five novel SNPs and two InDels that clearly differentiated the wild-type and mutant genotypes. Gene-based diversity using 11-InDel markers categorised 48-diverse maize-inbreds into two-clusters. The majority of mutant and wild-type inbreds were grouped in separate clusters and led to the generation of 41 haplotypes. Genetic relationship of maize *fatb* gene with orthologues among 40 accessions of 12 oilseed-crops using both nucleotide and protein sequence clustered maize, soybean, sunflower, opium-poppy, *Citrulus lanata*, quinoa, and prunus species into one cluster; and brassica, camelina, and arabidopsis into the different cluster. The clustering pattern revealed that the plant oil with higher unsaturated fatty acids, particularly oleic, linoleic, and linolenic acids grouped together in one cluster and higher proportions of other fractions like arachidic, eicosenoic, and erucic acids grouped in another cluster. Physico-chemical properties highlighted more similarity between maize and 29 orthologue proteins, but orthologues were found to have better thermostability. Homology models have been developed for maize mutant and wild-type inbreds using *Umbellularia californica* (PDB ID: 5x04) as a template. Predicted protein models possessed optimum confidence-score and RMSD values and validated stability via., Ramachandran plots. Molecular docking indicated most of the interactions of protein-ligand were having similar binding-affinity due to the broader specificity of fatty acyl-ACP thioesterases and the presence of conserved-domains across crops. This is the first report on the comprehensive molecular characterisation of the *fatb* gene in maize and various orthologues. The information generated here provided new insights into the genetic diversity of *fatb* gene which can be utilised for the enhanced nutritive value of oil in the breeding programme.

## Introduction

In plants, oil is stored in the form of triacylglycerols; which contains a limited number of fatty acids (1). Although oil seed crops enable humans to meet the daily requirement of vegetable oil, maize oil is gaining popularity as edible oil owing to its unique health benefits (2). Besides, maize is one of the most versatile crops grown in diverse ecologies; and serves as one of the most potential staple food crops capable of boosting global food security (3). Apart from its use as a food and feed, maize is a key source of vegetable oil due to the proper balance of saturated and unsaturated fatty acids thus oil quality (4).

Plant-based oils are primarily comprised of five common fatty acids, namely, palmitic acid (C_16:0_), stearic acid (C_18:0_), oleic acid (C_18:1_), linoleic acid (C_18:2_), and linolenic acid (C_18:3_) depending on the crop species (5). Excessive intake of saturated fatty acids (SFAs: palmitic and stearic acid) may lead to elevated blood cholesterol, triglycerides, and low-density lipoprotein cholesterol which in turn results in an increased risk of coronary heart diseases (6). On the other hand, unsaturated fatty acids (UFAs: oleic, linoleic, and linolenic acid) are beneficial to human health like oleic acid reduces the cholesterol problems in humans, linoleic acid contributes to anti-cancer properties and improved immunity, and linolenic acid decreases the risk of heart diseases (6, 7). Traditional maize grains possess ~3–4 % of oil, whereas high-oil maize constitutes more than 6% of oil (5). Maize oil is considered as a high-quality oil for human consumption, as it possesses 80.5% UFAs mostly of oleic acid (28.3%) and linoleic acid (50.6%), and 19.5% saturated fatty acids (SFAs), mostly of palmitic acid (12.1%) (4). The high stability of maize oil is attributed to high natural antioxidants (2, 8). Wide genetic variation (50 to 82%) for oleic acid has been reported in maize (9), indicating scope for improvement in the ratio of SFA to UFA in the commercial maize oil.

Thioesterases are the key enzymes determining the concentration and composition of fatty acids in oilseed crops (10). In plants, biosynthesis of fatty acids occurs in chloroplasts and a group of enzymes called acyl-ACP thioesterases catalyse the hydrolysis of acyl-ACP thioester bonds leading to chain termination, thereby releases free fatty acids and acyl carrier proteins (11). Later, free fatty acids are transported to cytosol and esterified by coenzyme-A and stored as triacylglycerols (TAGs). The evolutionary studies of *fatb* (*fatty acyl ACP-thioesterase b*) gene was first reported in Arabidopsis through development of T-DNA mutants, followed by *Brassica napus* through transgenic approaches (12, 13). There are two distinct but related thioesterase gene classes in higher plants, termed FatA and FatB, whose evolutionary divergence appears to be ancient. FatA encodes for 18:l-ACP thioesterase whereas, FatB representatives encode thioesterases preferring acyl-ACPs having saturated acyl groups (13). Type B fatty acyl-ACP thioesterase encoded by *fatb* gene has high affinity for saturated fatty acids as substrate (4, 14); especially for C_16:0_-ACP (12); and thus, the substrate preference determines the fatty acid composition in different crop plants.

Earlier researchers identified quantitative trait loci (QTLs) altering the fatty acid composition in maize (15–17). A major QTL-*Pal9* was identified on chromosome-9 explaining 42% of the phenotypic variation for palmitic acid in maize grains (16). Fine mapping of this major QTL led to identification of 4630 bp long *Zmfatb* gene which encodes acyl-ACP thioestersase; and an 11 bp insertion in the last exon of *Zmfatb* caused the reduction in palmitic acid, thereby optimising the ratio of SFAs to UFAs without affecting total oil content (7). The maximum change in palmitic acid was 4.57 mg/g, which accounted for 20 to 60% of the variation in the ratio of SFA to UFA (7). Further, Zheng et al. (4) reported insertion of single G-nucleotide in 6th exon of *Zmfatb*, which creates a premature stop codon leading to reduction (~60%) of palmitic acid content in maize. A total of 318 homologues including 218 orthologues and 10 paralogues for *fatb* and 30 homologues for *FAD* (fatty acid desaturase) genes were reported in maize (18, 19). No natural mutant source has been reported but evolutionary studies of *fatb* gene started with arabidopsis *fatb* T-DNA mutants and fatb transgenic *Brassica napus*. Down-regulation of *FatB* (involved in fatty acid synthesis) and *FAD2* (encoding Δ12 fatty acid desaturase) together prevented the conversion of oleic acid to polyunsaturated fatty acids (PUFA) and led to increased levels of oleic acid (85%) and low levels of SFAs (6%) in soybean (20, 21). Reducing the expression of seed-specific *FAD2* isogenes, *FAD2-1A* and *FAD2-1B* by gene silencing has led to release of high-oleic soybean varieties with oleic acid ranging from 72 to 77% (21, 22). Further, CRISPR/Cas-mediated genome editing for down regulation of *fatB1a* or *fatB1b* genes in soybean led to significant reduction of SFAs (6). In Brassica species, the *FAD2* genes are expressed in all plant tissues, particularly *Brassica napus* contains four isogenes for *FAD2* (*FAD2.A1, FAD2.A5, FAD2.C1*, and *FAD2.C5*). Mutations in these genes are well demonstrated to contribute to PUFA synthesis in all organs and tissues of the plant. By combining these mutations with conventional breeding approaches produced lines upto 85% of oleic acid in oil (21, 23). Recently, molecular docking studies using palmitic, stearic and oleic acids as substrates provided new insights into alteration of fatty acid composition in sunflower (24).

The genetic diversity for entire *Zmfatb* gene sequence among the diverse set of maize inbreds including high-oil lines have not been comprehensively analysed. Further, there is no report on allelic variation on the nucleotide diversity of full length *Zmfatb* gene affecting palmitic acid, and ratio of SFA to UFA. In addition, gene and protein sequence comparison of *fatb* among maize and its orthologues, especially oil seed crops based on oil composition are yet to be fully studied. Hence, the present investigation was designed to (i) sequence characterise *Zmfatb* gene in diverse maize mutant and wild-type maize inbreds, (ii) identify haplotypes of *Zmfatb* gene using gene-based InDel markers among diverse maize inbreds, (iii) study the evolutionary relationship of maize *fatb* with its orthologues at gene and protein level, and (iv) study the protein modelling and molecular docking in maize and its selected orthologues.

## Materials and Methods

### Genetic Material

Eight diverse wild type maize inbreds (*ZmfatB-*Wild1 to *ZmfatB-*Wild8) and two high-oil mutant inbreds (*Zmfatb-*Mutant1 and *Zmfatb-*Mutant2) were selected for sequence characterisation of full length *fatb* gene of maize at nucleotide and protein level (Table 1). The wild type allele is represented as ‘*ZmfatB*' and mutant allele as ‘*Zmfatb*'. A diverse panel of 48 genotypes including 27 wild-type and 21 mutant-type inbreds was used for assessing gene-based diversity and haplotype study (Supplementary Table 1).

**Table 1:** Details of diverse maize inbreds used in the study for characterisation of *fatb* gene.

**S. No**.	**Inbred**	**Code**	**Type**	**Source**
1.	PMI-Bio-101	*ZmfatB-*Wild1	Wild	ICAR-IARI, New Delhi
2.	PMI-Bio-102	*ZmfatB-*Wild2	Wild	ICAR-IARI, New Delhi
3.	PMI-Bio-103	*ZmfatB-*Wild3	Wild	ICAR-IARI, New Delhi
4.	PMI-Bio-104	*ZmfatB-*Wild4	Wild	ICAR-IARI, New Delhi
5.	EC932601	*ZmfatB-*Wild5	Wild	USDA
6.	EC932607	*ZmfatB-*Wild6	Wild	USDA
7.	CP828-2	*ZmfatB-*Wild7	Wild	ICAR-IIMR, Ludhiana
8.	CP828-1	*ZmfatB-*Wild8	Wild	ICAR-IIMR, Ludhiana
9.	EC932611-2	*Zmfatb-*Mutant1	Mutant	USDA
10.	EC932611-5	*Zmfatb-*Mutant2	Mutant	USDA

### Isolation of Genomic DNA, PCR Amplification and Gene Sequencing

The genomic DNA from seeds of selected inbreds was isolated using SDS extraction protocol (25). The mutant *Zmfatb* gene sequence of 4630 bp region from B73 genome (accession number: NC_050104 in Zm-B73-REFERENCE-NAM-5.0; GeneID: 103638097 or synonyms to GRMZM5G829544) was retrieved from NCBI data base. Eleven overlapping primer pairs designed using Primer3web v4.1.0 online tool (Supplementary Table 2) were synthesised from M/s Sigma Pvt. Ltd. The 11 primers covered full length *Zmfatb* gene with fragment size of 500-550 bp each. Each fragment was amplified using thermocycler in a 50 μl reaction consisting of 100 ng template DNA, 1x OnePCRTM Mix (GeneDireX Ready-to-use PCR master mix) and 0.5 μM each of forward and reverse primer. PCR amplification was carried out through BIO-RAD model T100TM thermal cycler (Bio-Rad Laboratories Inc.) with PCR conditions at (i) initial denaturation at 95°C for 5 min, (ii) 35 cycles of denaturation at 95°C, annealing at 60°C and primer extension at 72°C for 45 s each step and (iii) final extension at 72°C for 8 min. Each of the PCR reactions were carried out in replicates; each amplicon was checked on 2.0% Seakem LE agarose gel and the PCR products were processed for sequencing from M/s. Sequencher Pvt. Ltd.

### Sequence Alignment and Functional Analysis of *Zmfatb* Gene

Sequencing results of each fragment were blasted with B73 reference sequence on NCBI-Nucleotide BLAST to analyse raw sequence data. Whole gene sequence of each genotype was retrieved from Bio-Edit software by aligning 11 consecutive fragments covering whole gene. The whole gene sequences of all the genotypes along with B73 reference sequence were analysed in MEGA v7.0 tool using Clustal MUSCLE alignment to study SNPs and InDel variations among wild-type and mutant inbreds. The MEGA alignment file was then subjected to DnaSP6 v6.12.03 software to determine the number of SNPs, InDels, number of polymorphic sites, total number of mutations, haplotypes, haplotype gene diversity, nucleotide diversity and InDel events. The predicted mRNA sequence was used to study the synonymous and non-synonymous SNPs via. DnaSP6 v6.12.03 software. Putative SNPs clearly differentiating the mutants (*Zmfatb*) and wild-types (*ZmfatB*) were sorted manually. The functionality of SNPs in promoter region was determined using NsitePL (SOFTBERRY online programme) and SNPs in intron and polyA region were annotated through Ensembl Plants-Variant Effect Predictor (VEP) (26).

### *Zmfatb* Gene-Based Diversity Among the Diverse Genotypes

**Figure 1:**
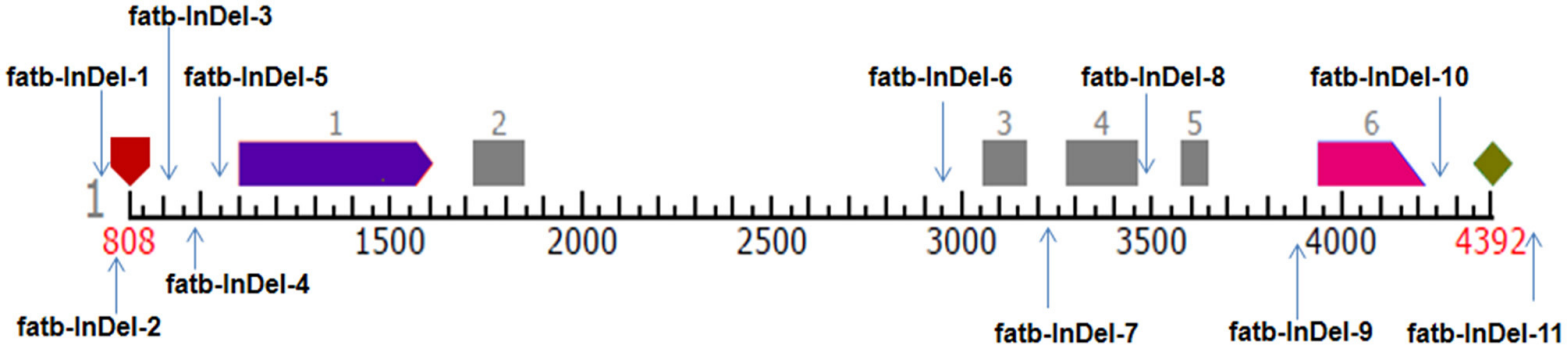
Pictorial representation of InDel-based markers position in *Zmfatb* gene. Pentagon in maroon colour represents TSS and rhombus in light green colour represents polyA site; light purple and pink arrows represent first and last exons; grey boxes represent exons.

Eleven InDel-based markers (Supplementary Table 3) were developed based on complete *Zmfatb* gene sequence among 10 genotypes used for sequencing and polymorphic positions were represented (Figure 1). PCR reactions and primer optimisation were done as per standard conditions. The banding pattern of each InDel marker among the diverse panel of 48 genotypes were obtained through gel electrophoresis using 4% metaphor agarose and 8% polyacrylamide gel electrophoresis (PAGE) based on fragment size. The obtained marker data based on gel profile was analysed in DARwin v6.0 to estimate genetic dissimilarity based on Jaccard's coefficient and constructed dendrogram using neighbour-joining method (27) and PowerMarker v3.25 to estimate parameters viz. total number of alleles, major allele frequency, gene diversity, heterozygosity and polymorphism information content (PIC) (28). Further, marker scores were exploited to generate haplotypes based on dendrogram clustering via. DARwin v6.0. The genotypes in single branch were considered as one haplotype. The presence of band is shaded in dark colour and absence of band is shaded in white colour to generate haplotypes among the diverse genotypes.

### Retrieval of *Zmfatb* Gene Sequence in Related Orthologues

Based on fatty acid composition, 12 orthologue crops including maize were selected; and the nucleotide and protein sequences were retrieved for different accessions of orthologue species. A total of 29 accessions namely, *Brassica napus* (5), *Brassica oleracea* (3), *Brassica rapa* (3), *Glycine max* (4), *Helianthus annuus* (4), *Citrulus lanatus* (2), *Camelina sativa* (3), *Chenopodium quinoa* (1), *Papaver somniferum* (1), *Prunus dulcis* (1), *Prunus persica* (1) and dicot model plant *Arabidopsis thaliana* (1) were obtained from publicly available data base via. Ensembl Plants using BLASTp search tool with an expectation value (e-value) ≤1e−5.

### Gene Prediction and Phylogenetic Relationship

All the 29 orthologue accessions along with 10 sequenced genotypes and B73 reference sequence of maize were employed individually to predict the number and size of exons and introns, 5' UTR, transcription start site, poly-A sites, mRNA sequence and protein sequence through online gene prediction software FGENESH (29). The complete gene sequence, predicted mRNA and protein sequences were aligned using CLUSTAL MUSCLE in MEGA v7.0 (30). Promoter component prediction was carried out using Plant-CARE online tool (31). A set of 40 *Zmfatb* sequences viz. (a) 10 sequences (8 *ZmfatB* and 2 *Zmfatb*) from the genotypes sequenced in the present study, (b) maize B73 reference sequence (*Zmfatb* mutant with GeneID: 103638097) available in public domain and (c) 29 orthologue accessions of related crop species (Supplementary Table 4) were considered for phylogenetic analysis for both nucleotide and protein sequences using online CLUSTAL MUSCLE tool. The genotypic score of each accession from CLUSTAL MUSCLE was further exploited through iTOL (Interactive tree of life) online software to construct dendrogram.

### Structural Analysis and Physicochemical Properties of ZmFATB Protein

Aligned nucleotide and protein sequences were manually sorted for presence of deletion, duplication, insertion, point mutation and conserved region. Transition-transversion bias, composition of nucleotide and Tajima's Neutrality test were under taken through MEGA v7.0 and DnaSP6 v6.11.01 (32). Chemical and physical parameters of protein like amino acids, molecular weight, isoelectric point (pI), positively and negatively charged amino acids and aliphatic index were estimated using MOTIF Search (https://www.genome.jp/tools/motif/) online tool. The instability index and grand average of hydropathicity (GRAVY) were calculated using ProtParam tool at ExPASy (33).

### Homology Modelling of ZmFATB Protein

At different complexity levels, ZmFATB protein homology models were built using SWISS-MODEL (34) and I-TASSER (35) automated protein structure homology-modelling servers. The best models were selected for further analysis based on various parameters like global model quality estimation (GMQE) score > 0.5, identity score > 50% with QMEAN score > 0.7. Stereo-chemical property of top predicted model was analysed through PROCHECK online server via. Ramachandran Plot.

### Sources of Ligands and Receptor Proteins

Plant oil is mainly composed of five compounds such as palmitic acid, stearic acid, oleic acid, linoleic acid and linolenic acid; and these substrates have been selected as ligands for the molecular docking studies in maize and its selected orthologues. The three-dimensional (3D) conformers of the selected ligands were retrieved from PubChem (https://pubchem.ncbi.nlm.nih.gov/) database in SDF format. Likewise, the protein sequence of the maize B73 reference (NP_001357940.1) and its selected orthologues viz. *Brassica napus* (A0A078GBF8), *Brassica oleracea* (Bo5g009040.1-1), *Brassica rapa* (Bra018620.1), soybean (KRH63043), sunflower (OTG24573), Arabidopsis (AT1G08510.1), *Camelina sativa* (Csa03g011960.1), *Citrulus lanatus* (Cla97C06G119690.1), quinoa (AUR62033409-RA), opium poppy (RZC53943) and almond (VVA10652) were subjected to SWISS-MODEL and I-TASSER servers to get homology models in PDB format for docking studies. The 3DLigandSite online server was employed to predict the ligand binding sites in protein structure (36).

### Preparation of Ligands and Target Proteins for Molecular Docking

The PDB files of each protein and the 3D structure of ligands were optimised using PyRx virtual screening software (37). The ligands were imported into PyRx through Open Babel version 3.1.1 (38), and charges of ligands were minimised and converted to PDBQT file format. Similarly, the protein structures in PDB were also converted to PDBQT files after optimisation.

### Molecular Docking and Visualisation

The optimised ligands and proteins in PDBQT format were subjected to molecular docking with the aid of the Vina Wizard tool in PyRx software (39). Vina wizard predicts the interaction between protein and ligand through its scoring function (binding affinity in kcal/mol). During molecular docking, based on predicted ligand binding sites, the grid box was adjusted to cover all the binding sites for each protein and XYZ coordinates were recorded. The default exhaustiveness value of 8 has been considered for docking. The output file after docking analysis consisted of the top nine binding poses with their binding affinity. The binding poses with the least root mean square deviation (RMSD) has been selected. The protein-ligand interaction was visualised in 3D through PyMOL software (40). Similarly, the 2D structure was visualised in Biovia Discovery studio (41). The 3D visualisation represents the exact binding site of the target protein whereas, the 2D structure visualisation represents the different bonds formed between amino acid residues of the target protein and the ligands.

## Results

### *Zmfatb* Gene Sequence Variation in Selected Maize Genotypes

The alignment of *Zmfatb* sequences revealed 1294 SNPs with nucleotide diversity (Pi) of 0.073. 540 InDels were also observed with a mean InDel length of 3.20 bp and InDels diversity (ki) of 56.55%. Nucleotide diversity (Pi) was found to be more in wild *ZmfatB* allele (0.08) compared to mutant *Zmfatb* allele (0.02) with an average nucleotide difference of 381.5 and 65.33, respectively. Tajimas's neutrality (D) value was −1.050004, which was inferred to be non-significant. The nucleotide sequence of the genotypes predicted (using MEGA software excluding the gaps and missing data with the final dataset of 4,310 bp) had an overall transition/transversion bias [R] of 0.7. In total, 3,412 bases were conserved among the selected 10 maize genotypes with sequence conservation [C] of 0.73. All the observed dynamic regions were found to be conserved significantly with a *p*-value < 0.01. The sequence characterisation provided 850 singleton variations with an average nucleotide frequency of 28.3 (T/U), 21.5 (C), 24.8 (A) and 25.4 (G) with 100% coverage of the *Zmfatb* gene sequence. The present investigation identified five SNPs and two InDels, which clearly differentiated the mutant (*Zmfatb*) and wild type (*ZmfatB*) genotype sequences (Table 2). Of these, SNP1 and InDel1 were in the promoter region; SNP2, SNP3 and SNP4 were in the intronic region, whereas SNP5 and InDel2 were found in the 3'UTR region. Both transition and transversion mutations occurred comparatively in similar frequency. Sequence analysis also validated the presence of 11 bp insertion in the 6th exon between mutant and wild-types, as reported by Li et al. (7).

**Table 2:** Putative SNPs and InDels clearly differentiate mutant and wild type maize inbreds.

**SNP**	**Type of mutation**	**Position (bp)**	**Location**	**Predicted function**
**SNPs**
SNP1^b^	C to G	663	Promoter	Cis-acting regulatory element
SNP2^a^	C to T	1,907	Intron	Intron variant
SNP3^a^	A to G	2,610	Intron	Intron variant
SNP4^b^	T to G	2,611	Intron	Intron variant
SNP5^a^	A to G	4,393	3'UTR	3'UTR variant
**InDel**	**Length (bp)**	**Position (bp)**	**Location**	**Predicted function**
**InDels**
InDel1	2	680–681	Promoter	Transcription factor binding site
InDel2	1	4,392	3'UTR	3'UTR variant

a *Transitions*;

b *Transversions*.

**Figure 2:**
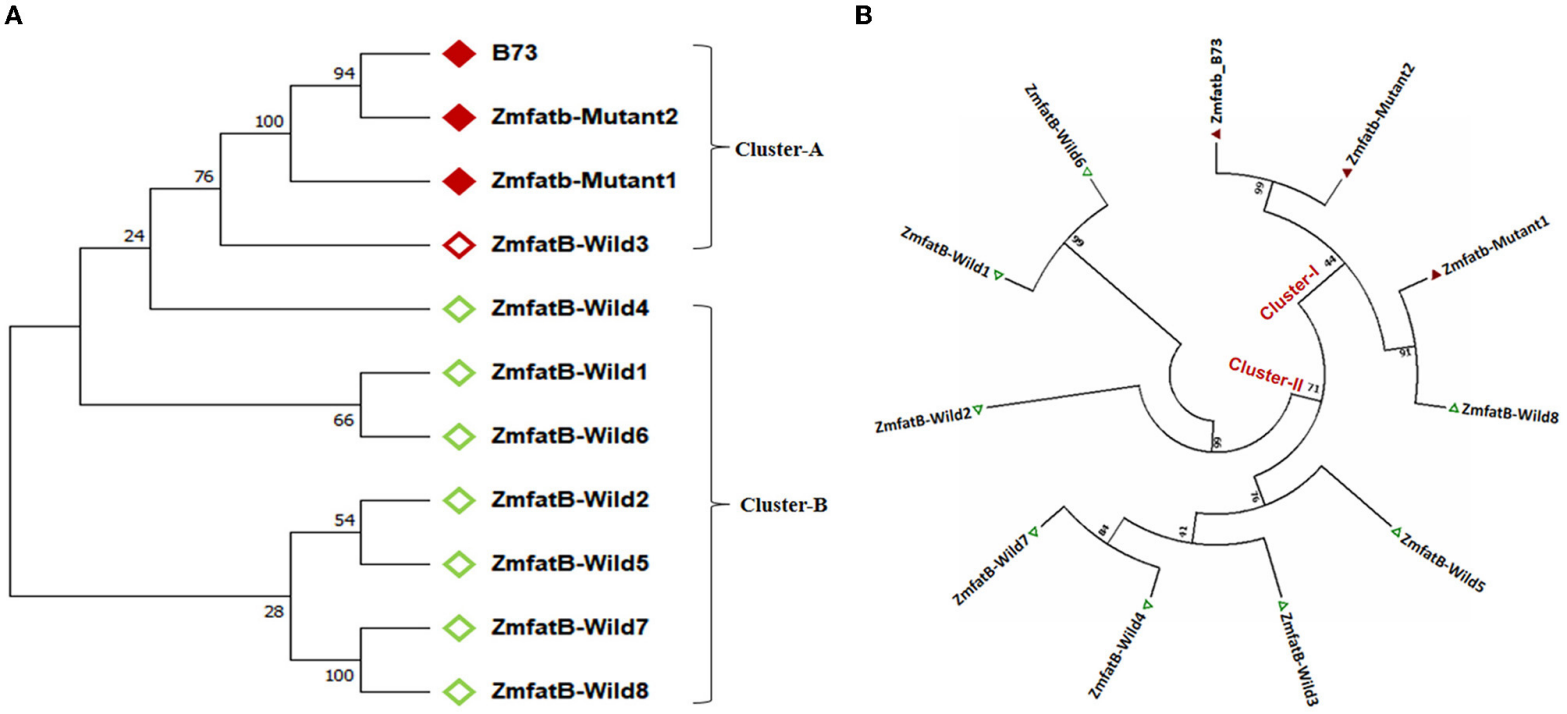
**(A)** Nucleotide based phylogenetic tree among the sequenced maize genotypes with 10000 bootstra p-value. **(B)** Protein-based phylogenetic tree among the sequenced maize genotypes with 10000 bootstra p-value.

Further, the *Zmfatb* sequence of these 10 maize genotypes were utilised for the construction of a phylogenetic tree along with the B73 reference sequence (acts as *Zmfatb* mutant) retrieved from the NCBI data base. The obtained tree consisted of two clusters namely -A and -B (Figure 2A). The mutant sequences viz. B73 (*Zmfatb*-mutant reference), *Zmfatb-*Mutant1 (EC932611-2) and *Zmfatb-*Mutant2 (EC932611-5), and *ZmfatB-*Wild3 (PMI-Bio-103) grouped together into cluster-A, and the cluster-B consisted of all other wild type genotypes. Clustering pattern predominantly differentiated the wild and mutant inbreds based on their sequences. The optimal tree with the sum branch length of 0.37 and strength of clustering at 10,000 bootstraps with associated taxa varied from 35 to 100% (Figure 2A).

The mRNA sequences have been generated (via. FGNESH online tool) for the 10 genotypes based on sequencing data and further employed to know the significance of observed polymorphism among the nucleotide sequences. An average of 51.02 synonymous (Ks) and 152.98 non-synonymous (Ka) sites were observed among the sequenced genotypes. Among the genotypes, *Zmfatb-*Mutant1 (EC932611-2), *Zmfatb-*Mutant2 (EC932611-5) and *ZmfatB-*Wild3 (PMI-Bio-103) had the Ka/Ks ratio of 0.000 indicating less significant changes in the coding region (Supplementary Table 5). Hence, these three genotypes grouped together in the same cluster. Among the wild type genotypes, *ZmfatB-*Wild6 (EC932607) had the highest Ka/Ks ratio (1.153) followed by *ZmfatB-*Wild2 (PMI-Bio-102) (0.95) and *ZmfatB-*Wild4 (PMI-Bio-104) (0.74) (Supplementary Table 5).

### Gene-Based Diversity Among the Diverse Maize Inbreds

Among the 540 InDels identified from *Zmfatb* sequence characterisation, selected 11 InDels (of 2 bp and more covering full-length gene) were exploited to develop the gene based InDel markers (Supplementary Table 3). The size of the InDels ranged from 2 to 33 bp and the primers were designed by considering the GC-content. A total of 25 alleles were generated with an allelic mean of 2.27 (range: 2 to 3) (Table 3). Major allele frequency varied from 0.56 (*fatb*-InDel-8) to 0.92 (*fatb*-InDel-2) with mean of 0.78. Mean genetic diversity observed was 0.33 (range: 0.15 to 0.49). PIC varied from 0.14 to 0.37 with a mean of 0.27. Among the 11 markers, five markers had a PIC of ≥ 0.3. Mean heterozygosity was 0.12 with a range of 0 to 0.63 (Table 3).

**Table 3:** Molecular diversity parameters among the 48 diverse genotypes using gene-based InDel markers.

**S. No**.	**Marker**	**Major allele frequency**	**No. of alleles**	**Gene diversity**	**Heterozygosity**	**PIC**
1.	*fatb*-InDel-1	0.91	3.00	0.17	0.10	0.17
2.	*fatb*-InDel-2	0.92	2.00	0.15	0.08	0.14
3.	*fatb*-InDel-3	0.86	2.00	0.23	0.10	0.21
4.	*fatb*-InDel-4	0.77	2.00	0.35	0.08	0.29
5.	*fatb*-InDel-5	0.79	2.00	0.33	0.08	0.28
6.	*fatb*-InDel-6	0.72	2.00	0.40	0.02	0.32
7.	*fatb*-InDel-7	0.77	3.00	0.37	0.00	0.34
8.	*fatb*-InDel-8	0.56	2.00	0.49	0.63	0.37
9.	*fatb*-InDel-9	0.79	2.00	0.33	0.00	0.28
10.	*fatb*-InDel-10	0.70	2.00	0.42	0.19	0.33
11.	*fatb*-InDel-11	0.78	3.00	0.35	0.06	0.30
Mean		0.78	2.27	0.33	0.12	0.27

*PIC, Polymorphism information content*.

**Figure 3:**
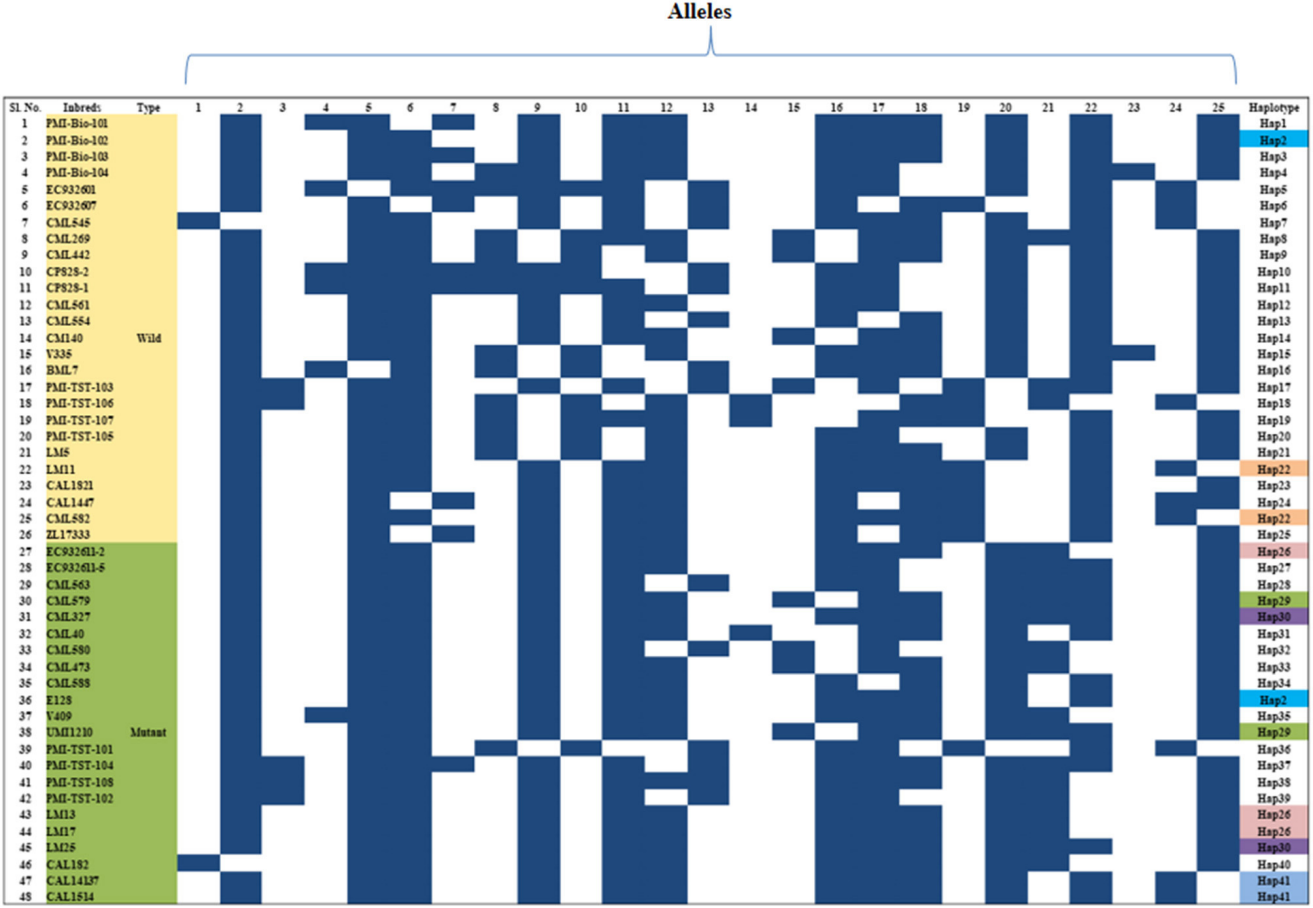
Haplotypes of *Zmfatb* gene using gene-based InDel markers; each row indicates the presence or absence of allele, whereas the column represents the allele for a given marker in diverse inbreds; Black box, presence of DNA band; White box, absence of DNA band.

The genetic relationship among the 48 genotypes was studied based on the dissimilarity matrix and the pair-wise genetic dissimilarity varied from 0 to 0.94 (mean: 0.42). Cluster analysis grouped them into two major clusters, namely -C and -D (Supplementary Figure 1). Twenty-eight genotypes were grouped in cluster-C, whereas cluster-D possessed 20 genotypes with two sub-clusters each in –C (C1 & C2) and –D (D1 & D2). Majority of the wild type genotypes were grouped into cluster-C and mutants were grouped into cluster-D. Clustering patterns also revealed that genotypes originated from similar/related pedigree grouped together (Supplementary Figure 1). Haplotype analysis based on marker-assay through InDel-based markers of *Zmfatb* revealed the presence of 41 haplotypes existed among the 48 inbreds (Figure 3).

### Phylogenetic Relationship Among *Zmfatb* Sequences of Maize and Selected Orthologues

**Figure 4:**
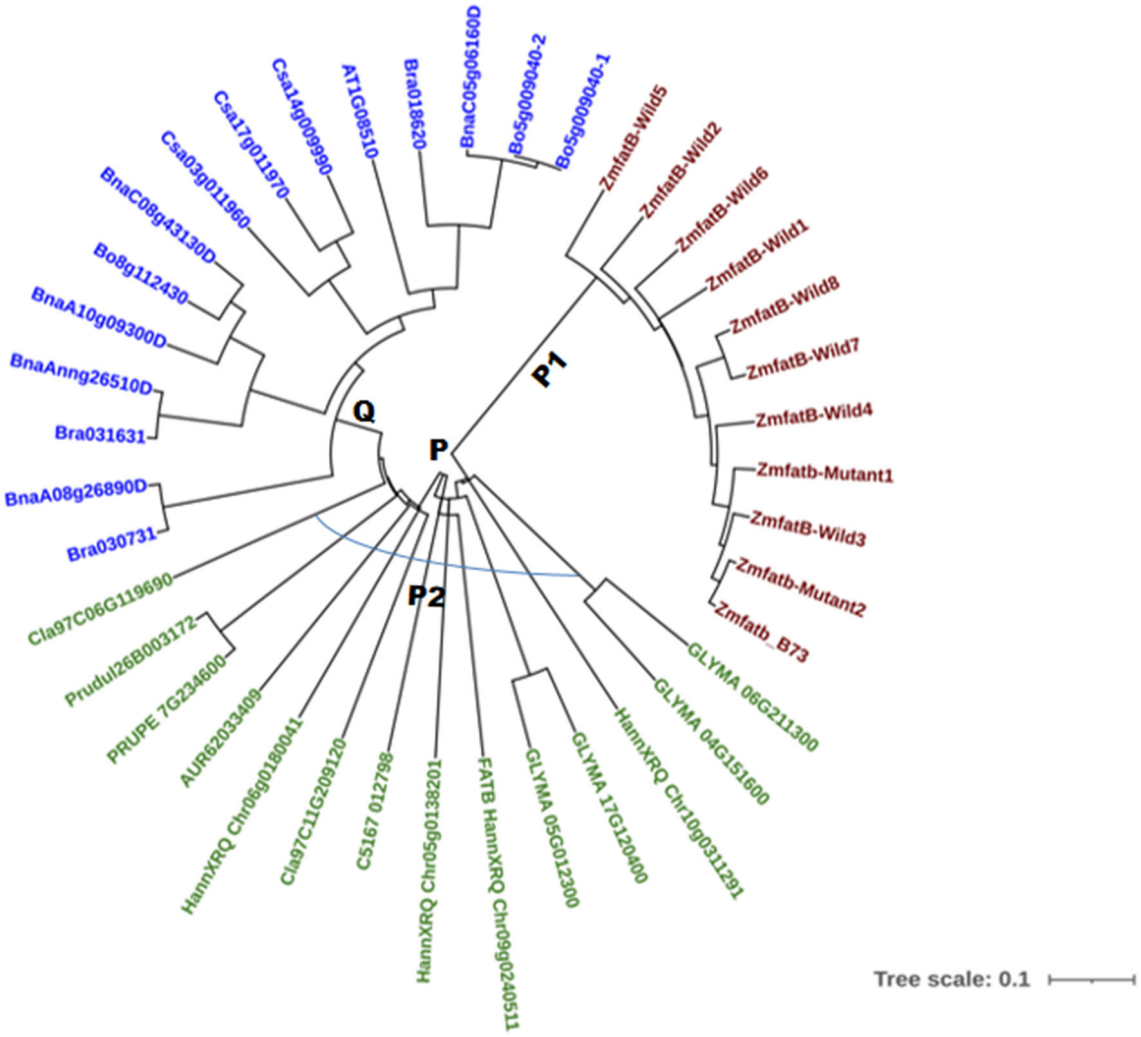
Nucleotide based phylogenetic tree of maize and its orthologue accessions.

A total of 40 accessions across 12 different crops were selected based on their fatty acid composition to study phylogenetic relationships; the details of the nucleotide and protein ID were presented in Supplementary Table 4. The phylogenetic tree was constructed using the neighbour joining method at 10000 bootstrap for both nucleotide and protein sequences. The phylogenetic tree based on nucleotide sequences of 40 accessions grouped them into two major clusters, namely -P and -Q (Figure 4). All the maize genotypes including B73 reference clustered into sub-cluster-P1. The accessions viz. soybean (GLYMA_04G151600, GLYMA_05G012300, GLYMA_06G211300, and GLYMA_17G120400), sunflower (HannXRQ_Chr05g0138201HannXRQ_Chr06g0180041, FATB_HannXRQ_Chr09g0240511 and HannXRQ_Chr10g0311291), Opium poppy (C5167_012798), *Citrullus lanatus* (Cla97C06G119690 and Cla97C11G209120), quinoa (AUR62033409) and *Prunus sp*. (Prudul26B003172 and PRUPE_7G234600) clustered into sub-cluster-P2. The remaining orthologue accessions viz. *Brassica napus* (BnaC08g43130D, BnaA08g26890D, BnaAnng26510D, BnaA10g09300D, and BnaC05g06160D), *Brassica oleracea* (Bo5g009040-1, Bo5g009040-2, and Bo8g112430), *Brassica rapa* (Bra018620, Bra030731 & Bra031631), *Camelina sativa* (Csa03g011960, Csa14g009990, and Csa17g011970) and *Arabidopsis thaliana* (AT1G08510) were grouped into cluster-Q (Figure 4). Clustering pattern clearly grouped the crops with higher UFA particularly oleic, linoleic and linolenic acids together and crops containing lower UFA clustered into another group.

To strengthen the clustering pattern of maize and the orthologues, the protein sequences were predicted and evolutionary tree was generated using the protein sequences. Clustering revealed the same pattern as that of nucleotide-based grouping (Supplementary Figure 2) with two main clusters (-R and -S). All the maize genotypes, soybean, sunflower, quinoa, almond, citrullus and opium poppy accessions grouped into cluster-R; and the accessions of all *Brassica sp.*, camelina and arabidopsis were grouped into cluster-S. Similar grouping pattern observed based on both nucleotide and protein levels suggested the conserved functional groups across the crops.

### *Zmfatb* Gene Structure in Maize and Its Orthologues

The sequence of *Zmfatb* in 10 maize genotypes and 29 accessions of orthologue retrieved from public domain were predicted for transcription start site (TSS), exon and intron boundaries, coding sequence, and polyA tail. The comparative analyses of all these parameters are presented in Supplementary Table 6. The analysis demonstrated that TSS for *Zmfatb* was found to be located 350-875 bp upstream in maize genotypes, whereas in orthologues, it is located 3-1,554 bp upstream of coding sequence start site. Total coding sequence varied from 924 to 1,275 bp among maize accessions and 1,191–1,914 bp among orthologues. Number of exons ranged from 4 to 8 in maize; whereas it varied from 5 to 8 among orthologues. Length of exons varied between 24 and 513 bp in maize, and in orthologues exon length varied between 24 and 768 bp. Wide intronic range was found in maize genotypes (50–1,749 bp) as compared to orthologues (77–1681 bp). PolyA site was located 4,350–4,498 bp downstream of coding start site in maize whereas, in orthologues it was located between 2,593 and 6,057 bp.

### ZmFATB Protein Characterisation Among Sequenced Genotypes

The complete nucleotide sequences of all the 10 maize genotypes along with B73 reference mutant were employed to predict the protein sequences. The comparison of protein sequences among wild and mutant inbreds revealed 285 variable regions with Tajimas's neutrality (D) value of 1.388231. There were 199 conserved amino acids among the genotypes with 201 singleton amino acid variations. In addition, the multiple sequence alignment also revealed insertion of five amino acids (SGVFR) in mutant genotypes from 425 to 428 amino acid position as reported earlier by Li et al. (7). The genotypes possessed overall mean amino acid difference of 0.382. Further, phylogenetic grouping based on protein sequence revealed existence of two clusters (-I and -II) with sum of branch length of 1.623. All the mutant genotypes including B73 were grouped together in cluster-I and all the wild type genotypes were grouped in cluster-II except *ZmfatB-*Wild8 (CP828-1) (Figure 2B).

### Prediction of Domains and Motifs of ZmFATB Protein

Domain prediction elucidated the existence of 8 conserved domains across the accessions for ZmFATB protein (Supplementary Table 7). PLN02370 superfamily domain was predicted to be bigger domain covering from 1 to 424 amino acids (aa) followed by pfam01643- acyl-ACP_TE domain (141 to 412 aa). Among the domains predicted, five domains viz. PLN02370 superfamily, pfam01643, pfam12590, FatA, and HotDog domain superfamily were found to be the major domains. Among all the maize and orthologue accessions, these five major domains were conserved except FatA domain in *ZmfatB-*Wild1 (PMI-Bio-101) and *Zmfatb-*Mutant2 (EC932611-5), as well as pfam01643 domain in sunflower accession (OTG12589).

### Physicochemical Properties of ZmFATB in Maize and Its Orthologues

Primary protein structure analysis revealed that the protein length was very diverse in maize accessions (307-434 amino acids) but in orthologues, the length was found to be relatively narrow (374 to 470 amino acids) (Table 4). Further, alanine was found to be the most abundant amino acid in maize genotypes (7.3 to 11.2%), but in orthologues, it was observed to be low (3.7–7.6%). Tyrosine was found to be the rarest (0.6–1.7% among maize genotypes; 1.2–2.3% among orthologues) amino acid. Molecular weight of orthologues accession of *Citrulus sp*. (Cla97C06G119690) was noticed to be the highest (52744.43 g/mol) among all the genotypes. All the accessions of maize and orthologues were found to have neutral to basic isoelectric point. The instability index among the maize genotypes varied from 31.90 [*ZmfatB-*Wild1 (PMI-Bio-101)] to 52.16 [*ZmfatB-*Wild6 (EC932607)] whereas, among the orthologues, it ranged from 27.31 (HannXRQ_Chr05g0138201) to 45.29 (Cla97C06G119690). GRAVY ranged from−0.227 to−0.554 among maize genotypes and−0.243 to−0.454 among orthologue accessions.

**Table 4:** Physico-chemical properties of ZmFATB protein in maize and its selected orthologue accessions.

**S. No**.	**Accessions**	**Crop**	**No. of amino acid**	**Molecular weight (g/mol)**	**Isoelectric point**	**Negatively charged aa (Asp + Glu)**	**Positively charged aa (Arg + Lys)**	**Instability index**	**Aliphatic index**	**GRAVY**
1.	*Zmfatb-* B73*-*Mutant	*Zea mays*	434	47760.39	9.01	46	51	35.20	80.07	−0.386
2.	*Zmfatb-*Mutant1	*Z. mays*	422	47820.82	8.82	46	51	41.43	80.66	−0.414
3.	*Zmfatb-*Mutant2	*Z. mays*	307	33151.41	9.41	31	37	38.71	73.81	−0.441
4.	*ZmfatB-*Wild1	*Z. mays*	411	44146.19	6.82	44	43	31.90	77.93	−0.283
5.	*ZmfatB-*Wild2	*Z. mays*	425	45936.78	8.87	39	45	44.42	81.76	−0.227
6.	*ZmfatB-*Wild3	*Z. mays*	429	47203.61	7.76	47	48	33.26	79.18	−0.381
7.	*ZmfatB-*Wild4	*Z. mays*	333	36475.38	9.68	32	42	36.85	70.72	−0.462
8.	*ZmfatB-*Wild5	*Z. mays*	395	43070.24	10.05	33	56	33.72	72.66	−0.496
9.	*ZmfatB-*Wild6	*Z. mays*	328	36332.32	10.44	29	46	52.12	71.16	−0.554
10.	*ZmfatB-*Wild7	*Z. mays*	325	35692.31	7.81	35	36	35.52	75.11	−0.424
11.	*ZmfatB-*Wild8	*Z. mays*	339	38301.59	8.28	42	44	35.93	79.44	−0.506
12.	BnaC08g43130D	*Brassica napus*	415	46054.22	7.66	48	49	38.97	77.49	−0.399
13.	BnaA08g26890D	*B. napus*	415	46218.56	8.71	47	51	36.99	78.84	−0.436
14.	BnaAnng26510D	*B. napus*	415	46038.24	8.18	47	49	36.55	77.25	−0.393
15.	BnaA10g09300D	*B.napus*	415	46054.22	7.66	48	49	38.97	77.49	−0.399
16.	BnaC05g06160D	*B. napus*	412	45778.29	8.62	46	49	41.44	81.31	−0.336
17.	Bo5g009040-1	*Brassica oleracea*	412	45778.29	8.62	46	49	41.44	81.31	−0.336
18.	Bo5g009040-2	*B. oleracea*	412	45778.29	8.62	46	49	41.44	81.31	−0.336
19.	Bo8g112430	*B. oleracea*	415	45997.17	6.98	48	48	38.62	78.43	−0.378
20.	Bra018620	*B.rapa*	412	45658.06	8.30	45	47	40.51	79.17	−0.350
21.	Bra030731	*Brassica rapa*	415	46266.73	8.70	47	51	35.02	80.24	−0.406
22.	Bra031631	*B. rapa*	415	45980.15	8.55	46	49	34.83	77.01	−0.399
23.	GLYMA_04G151600	*Glycine max*	422	46081.75	8.12	46	48	39.70	85.90	−0.245
24.	GLYMA_05G012300	*G. max*	416	45939.23	6.48	50	48	38.92	82.24	−0.357
25.	GLYMA_06G211300	*G. max*	419	46299.09	7.08	49	49	40.98	85.58	−0.277
26.	GLYMA_17G120400	*G. max*	416	45957.33	6.28	51	48	39.10	85.75	−0.326
27.	HannXRQ_Chr05 g0138201	*Helianthus annuus*	413	45950.28	6.77	51	50	27.31	82.06	−0.397
28.	HannXRQ_Chr06 g0180041	*H. annuus*	430	47658.00	7.10	55	55	33.00	76.35	−0.454
29.	FATB_HannXRQ_Ch r09g0240511	*H. annuus*	421	46668.15	7.01	50	50	34.58	81.24	−0.343
30.	HannXRQ_Chr10 g0311291	*H. annuus*	353	40458.85	9.04	41	48	40.46	86.60	−0.365
31.	Cla97C06G119690	*Citrulus lanatus*	479	52744.43	8.37	50	53	45.29	83.70	−0.256
32.	Cla97C11G209120	*C. lanatus*	469	52257.27	8.51	51	55	37.56	75.05	−0.348
33.	Csa03g011960	*Camellina sativa*	416	46096.58	8.75	46	50	38.97	77.74	−0.375
34.	Csa14g009990	*C. sativa*	416	46139.57	8.76	46	50	38.71	76.80	−0.385
35.	Csa17g011970	*C. sativa*	416	46109.54	8.76	46	50	37.67	76.80	−0.393
36.	AUR62033409	*Chenopodium quinoa*	423	46801.02	8.24	48	50	39.91	74.87	−0.451
37.	C5167_012798	*Papaver somniferum*	423	46894.32	8.19	42	44	36.71	84.61	−0.313
38.	Prudul26B003172	*Prunus dulcis*	417	45639.03	6.56	46	45	40.67	90.00	−0.247
39.	PRUPE_7G234600	*P. persica*	417	45594.98	6.56	46	45	41.06	89.54	−0.243
40.	AT1G08510	*Arabidopsis thaliana*	412	45687.07	8.76	46	50	35.99	80.39	−0.365

### Homology Modelling of ZmFATB Protein

**Figure 5:**
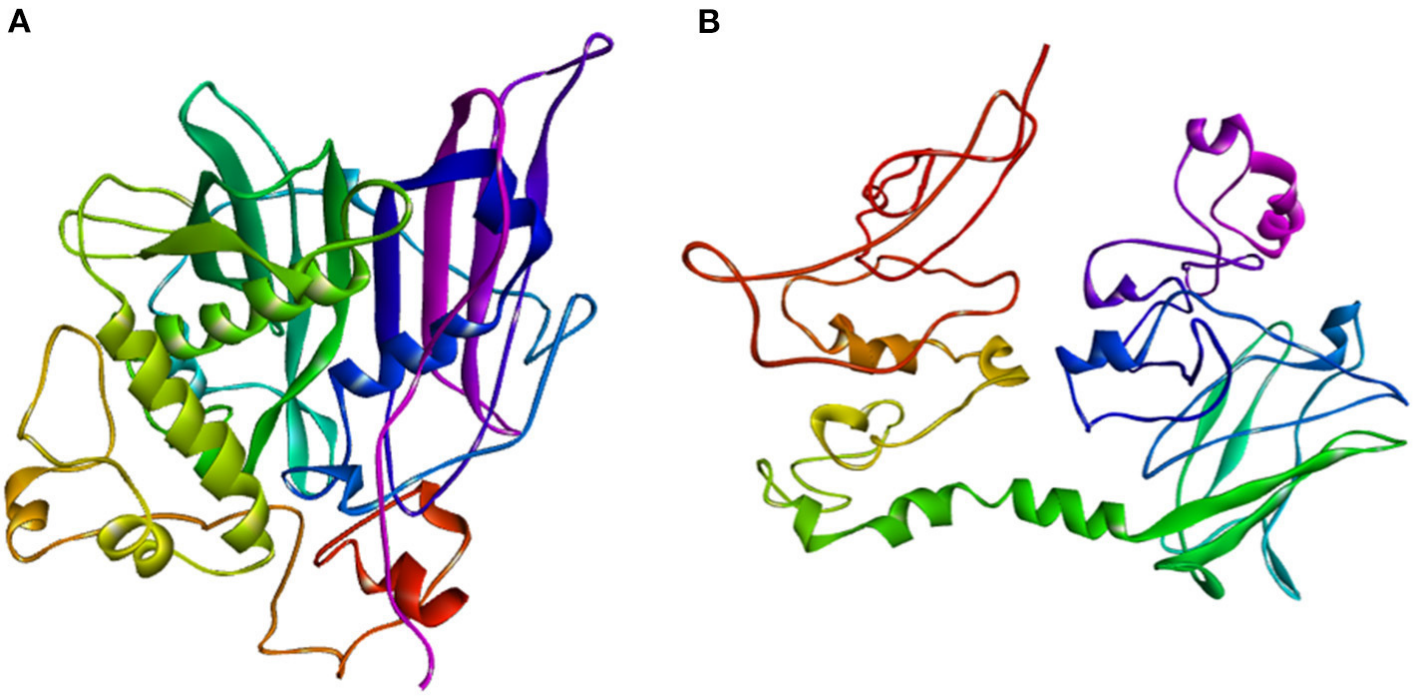
Thread based homology models of **(A)** mutant protein (ZmFATB-B73-Mutant); **(B)** wild type [ZmFATB-Wild1 (PMI-Bio-101)] protein obtained through I-TASSER.

The ZmFATB protein sequences of mutant and wild type maize inbreds were subjected to SWISS-MODEL and I-TASSER servers to identify similar templates. More than 500 templates were found to match with the target sequence of maize genotypes in SWISS-MODEL. Among the matched templates, 12:0-ACP thioesterase from *Umbellularia californica* (Protein Data Bank ID: 5xo4) which encodes for Dodecanoyl-[acyl-carrier-protein] hydrolase was found to have more similarity compared to others. The top protein model obtained through I-TASSER server was selected for further visualisation. Best model for B73 mutant had confidence (C)-score of−1.96, template modelling (TM)-score of 0.48 ± 0.15 and RMSD of 11.6 ± 4.5 A0, whereas the wild type maize inbred [*ZmfatB-*Wild1 (PMI-Bio-101)] had C-score of−2.34, TM-score of 0.44 ± 0.14 and RMSD of 12.5 ± 4.3 A0. These top models of B73 mutant reference and wild type (*ZmfatB-*Wild1) inbred were saved and visualised using Discovery studio 2020 (Figures 5A,B). Both B73 mutant reference and wild type (*ZmfatB-*Wild1) protein structures were superimposed through TM alignment and obtained superimposed protein structure with TM-score of 0.2419 and RMSD value of 7.23 (Supplementary Figure 3). Superimposition of both the proteins indicated that there is a random structural similarity between mutant and wild type proteins. Further, the stability of protein models was further checked through PROCHECK online server via. Ramachandran plot. The plots for both the models revealed that majority of residues (93.6 to 93.8%) lied in the most favoured region, 5.9 to 6% residues in the additional allowed region and a very small number of residues (0.4%) in generously allowed region and no residues in the disallowed region, indicating that superiority of obtained models (Supplementary Figures 4A,B).

### Protein and Ligand Interactions (Molecular Docking)

Molecular docking was carried out to determine the substrate specificity of FATB protein. The phylogenetic relationship among maize and orthologues species revealed that the accessions of maize, soybean, sunflower, *Citrulus sp.*, opium poppy, *Prunus sp*. and quinoa grouped in one cluster; and the accessions of other crops like Arabidopsis, *Brassica sp*. and *Camelina sativa* were grouped in another cluster. Therefore, one accession protein from each crop was considered to study the protein-ligand interaction using substrates like palmitic, stearic, oleic, linoleic and linolenic acids as ligands. The template from *Umbellularia californica* (PDB ID: 5xo4) was found to be the common template for protein for all the accessions and top model was saved in PDB format. The protein structure was found to have >50% similarity with template protein of *Umbellularia californica* (PDB ID: 5xo4) (Supplementary Table 8).

**Figure 6:**
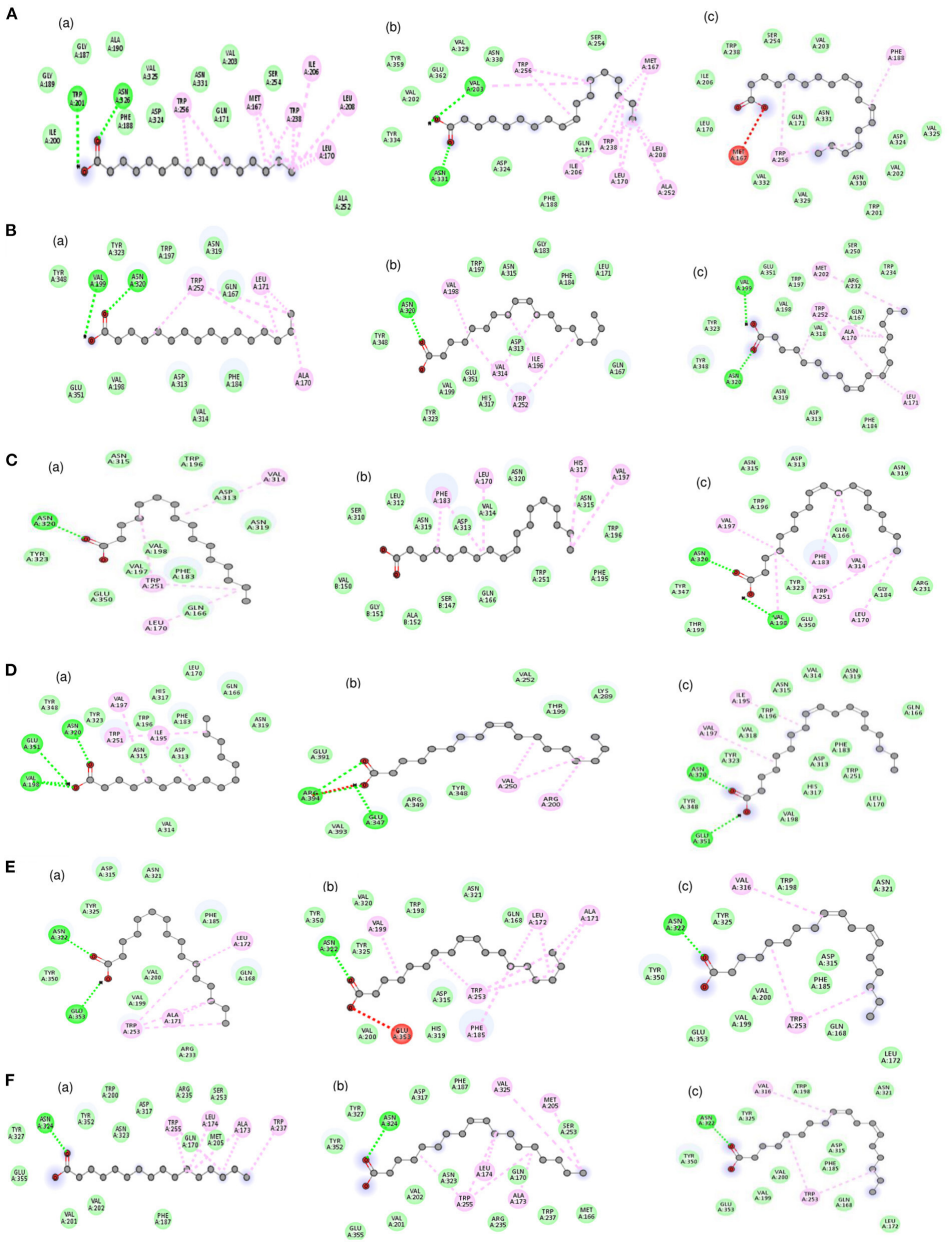
2D visualization of the interaction between protein and ligands. The various bonds formed between amino acid residues of various proteins with ligands have been depicted. Proteins accessions of **(A)** Maize B73 protein (NP_001357940.1); **(B)** Soybean (KRH63043); **(C)** Sunflower (OTG24573); **(D)** Arabidopsis (AT1G08510.1); **(E)** Brassica (A0A078GBF8); **(F)** Camelina (Csa03g011960.1). Ligands namely (a) Palmitic acid; (b) Oleic acid; (c) Linoleic acid. Bonds in green colour (hydrogen bond); red colour (Unfavorable acceptor-acceptor) and pink colour (Alkyl bond).

**Figure 7:**
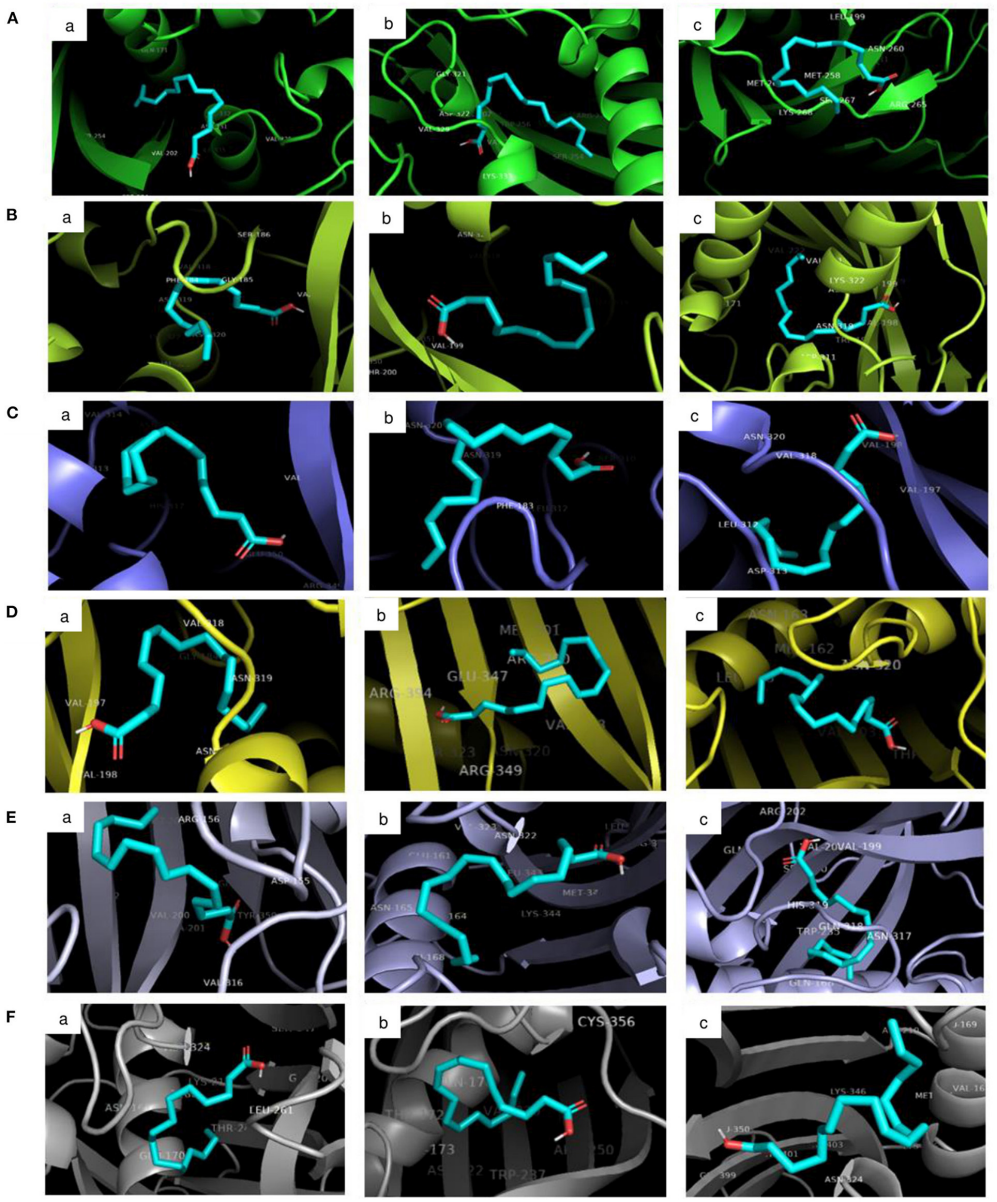
3D visualisation of the interaction between protein and ligands in PyMOL. The ligand-binding pose showing the highest binding affinity with the least root mean square deviation (RMSD) was selected. Proteins accessions of **(A)** Maize B73 protein (NP_001357940.1); **(B)** Soybean (KRH63043); **(C)** Sunflower (OTG24573); **(D)** Arabidopsis (AT1G08510.1); **(E)** Brassica (A0A078GBF8); **(F)** Camelina (Csa03g011960.1). Ligands namely (a) Palmitic acid; (b) Oleic acid; (c) Linoleic acid.

The molecular docking analysis of protein-ligand interaction among maize and orthologues species revealed that all the substrates bound to FATB protein with favourable binding energy ranging from −4 kcal/mol (oleic acid in Arabidopsis and linoleic acid in maize) to −7.3 kcal/mol (linoleic acid in maize) (Supplementary Table 8). Most of the protein-ligand interactions showed very good dock score above the threshold cut-off of−6 kcal/mol. When the binding energy of FATB protein was investigated for all the substrates, palmitic acid (−6.5 kcal/mol), stearic acid (−6.6 kcal/mol) and linolenic acid (−7.3 kcal/mol) in maize; oleic acid (−6.7 kcal/mol) in *Camelina sativa* and linoleic acid (−6.8 kcal/mol) in soybean exhibited highest binding affinity. Across the crops, the binding affinity was noticed to be almost similar with all the substrates. Among the crops, negligible variation was observed in binding affinity for different substrates. The results indicate that the substrates under study do not have much significant difference in most of the cases with respect to binding affinities across FATB proteins under consideration. However, difference was observed with respect to the hydrogen bond formation between substrates (Supplementary Table 9). The 2D and 3D visualisation of protein-ligand interactions are depicted in Figures 6, 7. The linoleic acid had highest binding affinity of−7.3 kcal/mol with two hydrogen bonds between VAL203 and ASN331 amino acid residues in maize. The substrate linolenic acid did not form hydrogen bond but possessing unfavourable acceptor-acceptor bond with MET167 amino acid residue in maize. The proteins of Arabidopsis and *Brassica rapa* formed three hydrogen bonds (amino acid residues VAL198, ASN320 and GLU351) with palmitic acid. Similarly, linoleic acid in *Prunus sp*. also formed three hydrogen bonds with amino acid residues SER248, TRP250 and ASN319. In most of the interactions, the amino acids like valine, aspergine and glutamic acids were found to form hydrogen bond with substrates (Supplementary Table 9; Figure 6).

## Discussion

Malnutrition is emerging as an alarming problem worldwide (42). Severe acute malnutrition (SAM) particularly affects more than 19 million children and contributes significantly to childhood morbidity and mortality (43). Reduced level of UFAs especially oleic and linoleic acid levels were considered to be associated with SAM, hence fatty acid composition plays critical role in human health. Considering the greater significance of *Zmfatb* gene in regulating the ratio of saturated to UFAs, we characterised the entire sequence of *Zmfatb* in 10 diverse maize inbreds to study allelic variations, gene-based diversity, phylogenetic relationships for *Zmfatb* in maize with orthologues present in oilseed crops, homology modelling and molecular docking of proteins to explore further possibilities of reducing the saturated fatty acids thereby improving the nutritional value of the oil.

### Allelic Variation for *Zmfatb* Among the Genotypes Sequenced

The sequence analysis revealed wide variation at SNP level within *Zmfatb* gene- an important factor to design better cultivars. Wide nucleotide diversity was found in wild type inbreds as compared to mutants; and this was due to their diverse origin (44, 45). This was also supported by Tajima's neutrality (D) value, where it was found to be significant in negative direction, indicating the selection pressure was due to lower average heterozygosity among the genotypes (46). The nucleotide sequence-based clustering pattern clearly indicated distinct variation between wild-type and mutants at nucleotide level for *Zmfatb* gene; hence both the mutants [*Zmfatb-*Mutant1 (EC932611-2) and *Zmfatb-*Mutant2 (EC932611-5)] and B73 reference gene (*Zmfatb*) grouped together into a single cluster. *Zmfatb* gene sequence of *Zmfatb-*Mutant2 was more similar to B73 reference than *Zmfatb-*Mutant1. Among the wild type inbreds, *ZmfatB-*Wild3 (PMI-Bio-103) had more similar *Zmfatb* sequence as that of mutants, hence grouped with the mutants. Evolutionary distance based on synonymous (Ks) and non-synonymous (Ka) sites is considered as powerful test of the neutral mode of evolution. The Ka/Ks ratio is used to infer the direction and magnitude of natural selection acting on protein coding genes and the ratio < 1 implies stabilising selection (47). Among the sequenced genotypes, all the inbreds possessed the Ka/Ks ratio of <1 except *ZmfatB-*Wild6 (EC932607) (1.153) indicating that the variation in terms of SNPs did not influence the protein expression. Further, the Ka/Ks ratio was found to be zero for both the mutants (*Zmfatb-*Mutant1 and *Zmfatb-*Mutant2) and *ZmfatB-*Wild3; hence they were grouped together into a single cluster.

The sequence alignment identified five novel SNPs and two InDels which clearly differentiated the wild and mutant type inbreds (Table 2). Among the SNPs, the frequencies of transitions were more (60%) compared to transversions (40%) similar to earlier reports (44, 48). Of the five SNPs, one SNP in promoter, three in intronic and one in 3' UTR region indicated the conservation of genomic regions among the genotypes. Occurrence of SNPs is found to be more frequent in non-coding regions than the coding regions as mutations do not affect the fitness of the gene (45, 49). The findings are further supported through protein sequence predicted using nucleotide sequence. The analysis revealed more conserved proteins as SNPs in non-coding region did not affect the coding regions. The clustering based on the protein sequence also grouped both the mutants along with maize B73 reference into one cluster.

### Molecular Characterisation and Haplotyping Using Gene-Based InDel Markers

Genetic diversity analyses based on gene-based markers enables the study of genetic relatedness and conservation of loci within the genes more efficiently. A total of 25 alleles were generated from 11 InDel markers. An average of 2.27 alleles/loci observed in the study was found to be higher as compared to Hossain et al. (50) (1.81 alleles/loci) and Chhabra et al. (45) (2.00 alleles/loci). Major allele frequency and PIC were comparable to earlier study by Hossain et al. (50) and Chhabra et al. (45). Lower mean heterozygosity was observed in the study which indicated that most loci were homozygous and the alleles were fixed. A total of 41 haplotypes for *Zmfatb* were generated in the study on the basis of InDels, which might be due to differential expression of acyl-ACP thioesterases among the genotypes. Among the haplotypes generated, Hap2 is found in PMI-Bio-102 and E128 inbreds, Hap22 in LM11 and CML582, Hap25 in EC932611-2, LM3 and LM17; and Hap41 in CML14137 and CAL1514. Chhabra et al. (45) identified 44 haplotypes among 48 genotypes based on *sugary1 (su1)* gene-based markers. Shin et al. (51) reported 14 haplotypes from 15 accessions of sweet corn using SNP based markers. Recent years have seen a surge in haplotype generation using sequencing technologies which can shed new light to trace ancestral history. The cost-effective and breeder friendly InDel-based markers from the present study would facilitate the identification of the *Zmfatb* based haplotypes among the unknown maize germplasm for its use in high oil breeding programme.

### Genetic Relationships Among Maize and Orthologues

The phylogenetic relationship based on nucleotide sequences among the 29 accessions from 11 oil seed crops and maize for *Zmfatb* grouped them into two clusters. The cluster-P included all maize, soybean, sunflower, opium poppy, quinoa, *Citrulus lanatus* and *Prunus sp*. accessions. The obtained results are well in agreement with the biochemical profile available in the literature. The fatty acid profile of the crops in cluster-P was found to possess more of unsaturated fatty acids (>80%) mainly includes oleic, linoleic and linolenic acids (4, 52–56). In the other cluster (Q), the accessions of *Brassica sp*., *Camelina sativa* and Arabidopsis grouped together and were found to have comparatively higher proportions of other fractions such as arachidic, eicosenoic acid and erucic acids (57–59). This clearly indicates that fatty acid biosynthesis pathway is considerably conserved among the plants, but significant variations for fatty acid content and composition is prevalent across the crop species (10, 57, 60). The clustering pattern based on protein sequence was similar to nucleotide-based grouping. Domain prediction indicated existence of eight domains for maize FATB protein (61); and of these domains, five domains namely PLN02370 superfamily, pfam01643, pfam12590, FatA and HotDog domain superfamily were found to be major and all the five domains were conserved across maize and orthologues except FatA domain in *ZmfatB-*Wild1 (PMI-Bio-101) and *Zmfatb-*Mutant2 (EC932611-5), and pfam01643 domain in sunflower accession (OTG12589). The conserved sequences across different crop species generate great significance in their functionality and evolution.

### Functional Characterisation of *Zmfatb* in Maize and Its Orthologues

The systematic efforts to trace the evolutionary origin of plant acyl-ACP thioesterases was reported by Jones et al. (12) and essential role of *fatb* was predicted through use of Arabidopsis *FATB* T-DNA mutants by Bonaventure et al. (13). To date, *fatb* gene has been characterised in majority of cereals and oil seed crops; but reports of *fatb* function in other plants are limited to few crops like Arabidopsis and maize (6). Through sequencing of *Zmfatb* among the 10 maize inbreds, the present study identified five novel SNPs (SNP1 to SNP5) and two InDels (InDel1 and InDel2) which can differentiate both wild and mutant types clearly. SNP1 is present in the promoter region; and it is responsible for G-box cis-acting regulatory element involved in the light responsiveness (31). InDel1 present in the promoter region, predicted to play a critical role in *opaque2* transcription factor binding site (TFBS) (29). Das et al. (49) also reported that sequence variations within promoter region have capacity to modify the binding of transcription factors thereby alter the functions. SNP2 to SNP4 were found in the intronic regions; the function prediction inferred that all these SNPs (SNP2 to SNP4) are acting as intron variants (modifiers). They may act on coding transcript, splice site variant, conserved intron variant or non-coding intron variants (26). SNPs matched with existing variant in Ensembl Plants like SNP2 matches with (PZE0920517496), SNP3 with PZE0920518199 and SNP4 with PZE0920518200 (26). SNP5 and InDel2 were present in 3'UTR; the function prediction determined that they act as modifiers causing 3'UTR variant regions. There are no matching variants for these regions and are found to be novel ones.

In addition, the sequence alignment of 10 sequenced genotypes, identified the presence of 11 bp insertion in the mutant genotypes and deletion in the wild type genotypes at 6^th^ exon of *Zmfatb* gene. Our results validated the findings of earlier report by Li et al. (7). The presence of 11 bp insertion leads to addition of five amino acids; thereby restricted the entry of substrate for palmitic acid production by making the portal smaller for substrate entry (7). The deletion of this region is associated with change in amino acids at its downstream. The orthologue species i.e., soybean possessed four homologues for *fatb* (GLYMA_04G151600, GLYMA_05G012300, GLYMA_06G211300 and GLYMA_17G120400). The first exon of soybean accession GLYMA_04G151600 had an insertion of 27 bp, other three accessions had 6 bp deletion (57). Cardinal et al. (60) determined that lines homozygous for the *fap*_*nc*_ mutation have a deletion in the *GmFatB* gene that encodes a 16:0-ACP thioesterase and it led to reduction in palmitic and stearic acid in their oil. An antisense and overexpression of *FATB* in Arabidopsis demonstrated that *in-vivo* production of palmitic acid in flowers and seeds (57). In same context, down regulation of *fatb* in soybean also demonstrated partial reduction of seed palmitic acid (62); but accessions with deletions in *fatb* gene had reduced palmitic acid content (60). Recently, Ma et al. (6) employed CRISPR/Cas-mediated genome editing technology to curtail the level of palmitic acid up to 39–53% in soybean.

### Features of FATB Protein in Maize and Orthologues

The ZmFATB protein is commonly called as palmitoyl-acyl carrier protein thioesterase, and it terminates the fatty acyl group extension via. hydrolysing an acyl group on a fatty acid; and occurrence of this protein in plant species is most common. The physico-chemical properties of FATB protein in maize and orthologues depicted that amino acid residues and molecular weight found to be the highest in *Citrulus lanatus* as compared to other orthologues; and it is due to longer coding sequences and more exons than others. Most of the accessions protein possessed isoelectric point (pI) of more than 8, which indicated that they were likely to precipitate in basic buffers and highly conserved functions (63). Aliphatic index signifies the thermo-stability of proteins; more the index, higher is the thermostability (64). Aliphatic index for maize genotypes is low as compared to orthologue accessions indicates the higher thermostability of orthologue proteins and they can sustain more temperature as compared to maize genotypes (63, 64). GRAVY represents the hydrophobicity value of a peptide; positive values indicate hydrophobic and negative values indicate hydrophilic nature of protein. GRAVY value (−0.227 to −0.554) in the study shows that all the proteins are hydrophilic in nature and has better interaction with water molecules.

The formulation of three-dimensional models enables the better understanding of the structural differences of the proteins since the general structure of these enzymes appeared to be conserved (24). Hence, homology modelling of ZmFATB protein in maize mutant (B73 reference) and wild type [*ZmfatB-*Wild1 (PMI-Bio-101)] was done to understand the structural differences among them. An available 12:0-ACP thioesterase crystal structure from *Umbellularia californica* as a template (PDB ID: 5X04) has been used for modelling. The protein sequence of B73 mutant and *Zmfatb-*Wild1 were 56.39% and 51.05% similarity with template (5xo4) protein. C-score is confidence for estimating the quality of predicted models by I-TASSER. A c-score of a higher value signifies a model with higher confidence (35). The protein of mutants had a better confidence score compared to wild type, indicating better quality of protein. TM-score and RMSD also represent the quality of the protein with the template. The mutants had better TM-score as compared to wild type, indicating better quality of protein in mutants, but both the protein structures possessed random similarities with the template proteins. Further, superimposition of both mutant and wild types through TM-align indicated the random structural similarity of the proteins with each other. Since, the insertion of 11 bp (7) and G- nucleotide (4) at the 6^th^ exon in *Zmfatb* mutants have been reported; this might be the reason for the random structural similarity between mutant and wild type proteins. The stability of these models was checked through PROCHECK online server and it indicated the superiority of both the models (mutant and wild type) (65).

Further, a molecular docking study among maize and orthologues revealed that most of the interactions were having docking scores of more than the threshold cut-off of −6 kcal/mol (66). The highest score of −7.30 kcal/mol for the interaction of linoleic acid with maize protein suggests that it requires less energy to bind with protein. Across the crop species, mostly similar binding affinity was observed with all the substrates and among the crops, slight variation was observed in binding affinity for different substrates. Since *fatb* gene displayed a broad specificity profile, showing similar activity to all the substrates (67, 68). This might be the probable reason for having similar dock scores. But hydrogen bond energy is the major contributor to dock score and it reflects the firmness of bonding between the protein and ligand (66). The formed hydrogen bonds between the protein and substrate can be employed to modify the fatty acid composition since major conserved domains have been observed across the crop species. The conserved domains consist of various acyl-ACP thioesterases and these terminate the acyl group extension by hydrolysing an acyl group (11). This might generate new insights into the modification of fatty acid composition by targeting binding pockets of amino acid residues specific to each substrate thereby improving the nutritional value of the oil.

## Conclusions

Characterisation of full-length *Zmfatb* gene among diverse maize inbreds revealed that five novel SNPs and two InDels differentiated the wild and mutant genotypes. This would serve as a potential region(s) to develop markers for the *Zmfatb* gene. Gene-based diversity using InDel markers identified 41 haplotypes of *Zmfatb* among the 48 diverse genotypes. The newly developed gene-based InDel markers can act as a cost-effective and friendly tool to characterise the unknown genotypes for fatty acids. Physico-chemical properties of maize and orthologues indicated that most of the crop accessions were having nearly similar molecular weight and precipitation in basic buffers. Orthologue proteins are more thermostable than maize proteins with similar hydrophilic nature of proteins. Most of the interactions showed similar binding affinity due to the broader specificity of fatty acyl-ACP thioesterases. The information generated in the study would provide new insights into the modifications of fatty acid composition, thereby enhancing the nutritive value of the oil and combating the issue of malnutrition.

## Data Availability Statement

The original contributions presented in the study are included in the article/Supplementary Materials, further inquiries can be directed to the corresponding author.

## Author Contributions

VM, FH, and DY: contributed to conception and design of the study. AK: conduct of the experiment. AK and RC: designing and assay of markers. AK and SM: *in-silico* data generation. AK and RZ: allelic diversity analysis. VM, FH, and RZ: development and maintenance of mutant inbreds. AK, VM, FH, and DY: drafting and editing of manuscript. All authors contributed to manuscript revision, read, and approved the submitted version.

## Funding

This study was supported by the Indian Council of Agricultural Research (ICAR) sponsored Consortia Research Platform on Biofortification in Selected Crops for Nutritional Security-Maize. Authors also acknowledge the support received from the World Bank-Indian Council of Agricultural Research funded National Agricultural Higher Education Project (NAHEP) through its Centre for Advanced Agricultural Science and Technology (CAAST) on Genomics Assisted Breeding for Crop Improvement to ICAR-IARI, New Delhi.

## Conflict of Interest

The authors declare that the research was conducted in the absence of any commercial or financial relationships that could be construed as a potential conflict of interest. The reviewer AKS declared a shared affiliation with the authors to the handling editor at the time of review.

## Publisher's Note

All claims expressed in this article are solely those of the authors and do not necessarily represent those of their affiliated organizations, or those of the publisher, the editors and the reviewers. Any product that may be evaluated in this article, or claim that may be made by its manufacturer, is not guaranteed or endorsed by the publisher.

## References

[1] Zhang X Wei W Tao G Jin Q Wang X. Identification and quantification of triacylglycerols using ultraperformance supercritical fluid chromatography and quadrupole time-of-flight mass spectrometry: Comparison of human milk, infant formula, other mammalian milk, and plant oil. J Agric Food Chem. (2021) 69:8991–9003. 10.1021/acs.jafc.0c07312 33755452

[2] Li H Thrash A Tang JD He L Yan J Warburton ML. Leveraging GWAS data to identify metabolic pathways and networks involved in maize lipid biosynthesis. Plant J. (2019) 98:853–63. 10.1111/tpj.14282 30742331 PMC6850169

[3] Prasanna BM Palacios-Rojas N Hossain F Muthusamy V Menkir A Dhliwayo T. Molecular breeding for nutritionally enriched maize: status and prospects. Front Genet. (2020) 10:1392. 10.3389/fgene.2019.01392 32153628 PMC7046684

[4] Zheng P Babar MD Parthasarathy S Gibson R Parliament K Flook J. A truncated *FatB* resulting from a single nucleotide insertion is responsible for reducing saturated fatty acids in maize seed oil. Theor. Appl Genet. (2014) 127:1537–47. 10.1007/s00122-014-2317-8 24802074

[5] Lambert RJ. High-oil corn hybrids. In Hallau AR editors. Special Corn. Boca Raton: CRC Press. (2001). p. 131–53. 10.1201/9781420038569.ch5

[6] Ma J Sun S Whelan J Shou H. CRISPR/Cas9-mediated knockout of GmFATB1 significantly reduced the amount of saturated fatty acids in soybean seeds. Int J Mol Sci. (2021) 22:3877. 10.3390/ijms22083877 33918544 PMC8069101

[7] Li L Li H Li Q Yang X Zheng D Warburton M. An 11-bp insertion in *Zea mays fatb* reduces the palmitic acid content of fatty acids in maize grain. PLoS ONE. (2011) 6:e24699. 10.1371/journal.pone.0024699 21931818 PMC3172307

[8] Zhang X Hong M Wan H Luo L Yu Z Guo R. Identification of key genes involved in embryo development and differential oil accumulation in two contrasting maize genotypes. Genes. (2019) 10:993. 10.3390/genes10120993 31805727 PMC6947151

[9] Wright A. A gene conditioning high oleic maize oil, *OLC1*. Maydica. (1995) 40:85–8.

[10] Voelker T. Plant acyl-ACP thioesterases: chain-length determining enzymes in plant fatty acid biosynthesis. Genet Eng. (1996) 18:111–33. 10.1007/978-1-4899-1766-9_8 8785117

[11] Yuan L Voelker TA Hawkins DJ. Modification of the substrate specificity of an acyl-acyl carrier protein thioesterase by protein engineering. Proc Natl Acad Sci USA. (1995) 92:10639–43. 10.1073/pnas.92.23.10639 7479856 PMC40667

[12] Jones A Davies HM Voelker TA. Palmitoyl-acyl carrier protein (ACP) thioesterase and the evolutionary origin of plant acyl-ACP thioesterases. Plant Cell. (1995) 7:359–71. 10.1105/tpc.7.3.359 7734968 PMC160788

[13] Bonaventure G Salas JJ Pollard MR Ohlrogge JB. Disruption of the *FATB* gene in Arabidopsis demonstrates an essential role of saturated fatty acids in plant growth. Plant Cell. (2003) 15:1020–33. 10.1105/tpc.008946 12671095 PMC152346

[14] Salas JJ Ohlrogge JB. Characterization of substrate specificity of plant FatA and FatB acyl-ACP thioesterases. Arch Biochem Biophys. (2002) 403:25–34. 10.1016/S0003-9861(02)00017-6 12061798

[15] Belo A Zheng P Luck S Shen B Meyer DJ Li B. Whole genome scan detects an allelic variant of *fad2* associated with increased oleic acid levels in maize. Mol Genet Genom. (2008) 279:1–10. 10.1007/s00438-007-0289-y 17934760

[16] Yang X Guo Y Yan J Zhang J Song T Rocheford T. Major and minor QTL and epistasis contribute to fatty acid compositions and oil concentration in high-oil maize. Theor Appl Genet. (2010) 120:665–78. 10.1007/s00122-009-1184-1 19856173

[17] Li H Peng Z Yang X Wang W Fu J Wang J. Genome-wide association study dissects the genetic architecture of oil biosynthesis in maize kernels. Nat Genet. (2013) 45:43–50. 10.1038/ng.2484 23242369

[18] Kersey PJ Allen JE Armean I Boddu S Bolt BJ Carvalho-Silva D. Ensembl genomes 2016: more genomes, more complexity. Nucleic Acids Res. (2016) 44:D574–80. 10.1093/nar/gkv1209 26578574 PMC4702859

[19] Zhao X Wei J He L Zhang Y Zhao Y Xu X. Identification of fatty acid desaturases in maize and their differential responses to low and high temperature. Genes. (2019) 10:445. 10.3390/genes10060445 31210171 PMC6627218

[20] Heppard EP Kinney AJ Stecca KL Miao GH. Developmental and growth temperature regulation of two different microsomal [omega]-6 desaturase genes in soybeans. Plant Physiol. (1996) 110:311–9. 10.1104/pp.110.1.311 8587990 PMC157722

[21] Bai S Engelen S Denolf P Wallis JG Lynch K Bengtsson JD. Identification, characterization and field testing of *Brassica napus* mutants producing high-oleic oils. Plant J. (2019) 98:33–41. 10.1111/tpj.14195 30536486 PMC6604813

[22] Wilson RF. The role of genomics and biotechnology in achieving global food security for high-oleic vegetable oil. J Oleo Sci. (2012) 61:357–67. 10.5650/jos.61.357 22790166

[23] Chalhoub B Denoeud F Liu S Parkin IA Tang H Wang X. Early allopolyploid evolution in the post-Neolithic *Brassica napus* oilseed genome. Sci. (2014) 345:950–3. 10.1126/science.1253435 25146293

[24] Aznar-Moreno JA Sanchez R Gidda SK Martinez-Force E Moreno-Perez AJ Caleron MV. New insights into sunflower (Helianthus annuus L) FatA and FatB thioesterases, their regulation, structure and distribution. Front Plant Sci. (2018) 9:1496. 10.3389/fpls.2018.01496 30459777 PMC6232763

[25] Dellaporta SL Wood J Hicks JB. Maize DNA miniprep. In: Malberg R Messing J Sussex I editors. Molecular Biology of Plants. New York, NY: Cold Spring Harbor (1985). p. 36–7.

[26] McLaren W Gil L Hunt SE Riat HS Ritchie GR Thormann A. The ensembl variant effect predictor. Genome Biol. (2016) 17:122. 10.1186/s13059-016-0974-4 27268795 PMC4893825

[27] Perrier X Flori A Bonnot F. Data analysis methods. In Hamon P Seguin M Perrier X Glaszmann JC editors. Genetic Diversity of Cultivated Tropical Plants. New York, NY: CRC Press (2003). p. 43–76.

[28] Liu K Muse SV. PowerMarker: integrated analysis environment for genetic marker data. Bioinform. (2005) 21:2128–9. 10.1093/bioinformatics/bti282 15705655

[29] Solovyev V Kosarev P Seledsov I Vorobyev D. Automatic annotation of eukaryotic genes, pseudogenes and promoters. Genome Biol. (2006) 7:1–2. 10.1186/gb-2006-7-s1-s10 16925832 PMC1810547

[30] Kumar S Stecher G Tamura K. MEGA7: molecular evolutionary genetics analysis version 7.0 for bigger datasets. Mol Biol Evol. (2016) 33:1870–4. 10.1093/molbev/msw054 27004904 PMC8210823

[31] Lescot M Dehais P Moreau Y De Moor B Rouze P Rombauts S. PlantCARE: a database of plant cis-acting regulatory elements and a portal to tools for in silico analysis of promoter sequences. Nucleic Acids Res. (2002) 30:325–7. 10.1093/nar/30.1.325 11752327 PMC99092

[32] Rozas J Ferrer-Mata A Sanchez-DelBarrio JC Guirao-Rico S Librado P Ramos-Onsins SE. DnaSP6: DNA sequence polymorphism analysis of large data sets. Mol Biol Evol. (2017) 34:3299–302. 10.1093/molbev/msx248 29029172

[33] Gasteiger E Hoogland C Gattiker A Duvaud S Wilkins MR Appel RD. Protein identification and analysis tools on the ExPASy Server. In Walker JM editor. The Proteomics Protocols Handbook. Totowa, NJ: Humana Press (2005). p. 571–607. 10.1385/1-59259-890-0:571

[34] Waterhouse A Bertoni M Bienert S Studer G Tauriello G Gumienny R. SWISS-MODEL: homology modelling of protein structures and complexes. Nucleic Acids Res. (2018) 46:W296-303. 10.1093/nar/gky427 29788355 PMC6030848

[35] Yang J Zhang Y. I-TASSER server: new development for protein structure and function predictions. Nucleic Acids Res. (2015) 43:W174–81. 10.1093/nar/gkv342 25883148 PMC4489253

[36] Wass MN Kelley LA Sternberg MJ. 3DLigandSite: predicting ligand-binding sites using similar structures. Nucleic Acids Res. (2010) 38:W469–73. 10.1093/nar/gkq406 20513649 PMC2896164

[37] Dallakyan S Olson AJ. Small-molecule library screening by docking with pyrx. Methods Mol Biol. (2015) 1263:243–50. 10.1007/978-1-4939-2269-7_19 25618350

[38] O'Boyle NM Banck M James CA Morley C Vandermeersch T Hutchison GR. Open Babel: an open chemical toolbox. J Chem Inf Model. (2011) 3:33. 10.1186/1758-2946-3-33 21982300 PMC3198950

[39] Trott O Olson AJ. AutoDock Vina: improving the speed and accuracy of docking with a new scoring function, efficient optimization, and multithreading. J Comput Chem. (2010) 31:455–61. 10.1002/jcc.21334 19499576 PMC3041641

[40] DeLano WL. The PyMOL Molecular Graphics System. (2009). Available online at: http://www.pymol.org.

[41] Biovia DS. Discovery Studio Visualizer, Release 2020. San Diego, CA: Dassault Systemes (2020).

[42] Virk PS Andersson MS Arcos J Govindaraj M Pfeiffer WH. Transition from targeted breeding to mainstreaming of biofortification traits in crop improvement programs. Front Plant Sci. (2021) 12:703990. 10.3389/fpls.2021.703990 34594348 PMC8477801

[43] Black RE Victora CG Walker SP Bhutta ZA Christian P De Onis M. Maternal and child undernutrition and overweight in low-income and middle-income countries. Lancet. (2013) 382:427–51. 10.1016/S0140-6736(13)60937-X 23746772

[44] Zunjare RU Chhabra R Hossain F Baveja A Muthusamy V Gupta HS. Molecular characterization of 5′ UTR of the *lycopene epsilon cyclase (lcyE)* gene among exotic and indigenous inbreds for its utilization in maize biofortification. 3 Biotech. (2018) 8:1–9. 10.1007/s13205-018-1100-y 29354386 PMC5766451

[45] Chhabra R Muthusamy V Gain N Katral A Prakash NR Zunjare RU. Allelic variation in *sugary1* gene affecting kernel sweetness among diverse mutant- and wild- type maize inbreds. Mol Genet Genom. (2021) 296:1085–102. 10.1007/s00438-021-01807-9 34159441

[46] Tajima F. Statistical method for testing the neutral mutation hypothesis by DNA polymorphism. Genet. (1989) 123:585–95. 10.1093/genetics/123.3.585 2513255 PMC1203831

[47] Yang Z Bielawski JP. Statistical methods for detecting molecular adaptation. Trends Ecol Evol. (2000) 15:496–503. 10.1016/S0169-5347(00)01994-7 11114436 PMC7134603

[48] Vignesh M Nepolean T Hossain F Singh AK Gupta HS. Sequence variation in 3′UTR region of *crtRB1* gene and its effect on β-carotene accumulation in maize kernel. J Plant Biochem Biotechnol. (2013) 22:401–8. 10.1007/s13562-012-0168-4

[49] Das AK Chhabra R Muthusamy V Chauhan HS Zunjare RU Hossain F. Identification of SNP and InDel variations in the promoter and 5′ untranslated regions of γ*-tocopherol methyl transferase (ZmVTE4)* affecting higher accumulation of α-tocopherol in maize kernel. Crop J. (2019) 7:469–79. 10.1016/j.cj.2019.01.004

[50] Hossain F Chhabra R Devi EL Zunjare RU Jaiswal SK Muthusamy V. Molecular analysis of mutant granule-bound starch synthase-I (waxy1) gene in diverse waxy maize inbreds. 3 Biotech. (2019) 9:1–10. 10.1007/s13205-018-1530-6 30555769 PMC6289911

[51] Shin JH Kwon SJ Lee JK Min HK Kim NS. Genetic diversity of maize kernel starch-synthesis genes with SNAPs. Genome. (2006) 49:1287-96. 10.1139/g06-116 17213911

[52] Sengupta A Mazumder UK. Triglyceride composition of *Papaver somniferum* seed oil. J Sci Food Agric. (1976) 27:214–8. 10.1002/jsfa.2740270303 1263455

[53] Lawal SA Choudhury IA Nukman Y. An assessment of the physico-chemical properties of melon seed (*Citrullus lanatus*) oil as base material for oil-in-water emulsion cutting fluid. Adv Mat Res. (2012) 576:293–5. 10.4028/www.scientific.net/AMR.576.293

[54] Wejnerowska G Ciaciuch A. Optimisation of oil extraction from quinoa seeds with supercritical carbon dioxide with co-solvents. Czech J Food Sci. (2018) 36:81–7. 10.17221/122/2017-CJFS

[55] Abdelghany AM Zhang S Azam M Shaibu AS Feng Y Qi J. Natural variation in fatty acid composition of diverse world soybean germplasms grown in China. Agron. (2019) 10:24. 10.3390/agronomy10010024

[56] Ouzir M Bernoussi SE Tabyaoui M Taghzouti K. Almond oil: A comprehensive review of chemical composition, extraction methods, preservation conditions, potential health benefits, and safety. Compr Rev Food Sci Food Saf . (2021) 20:3344–87. 10.1111/1541-4337.12752 34056853

[57] Sharma A Chauhan R. In silico identification and comparative genomics of candidate genes involved in biosynthesis and accumulation of seed oil in plants. Comp Funct Genomics. (2012) 2012:914843. 10.1155/2012/914843 22312320 PMC3270531

[58] Cartea E Haro-Bailon D Padilla G Obregon-Cano S del Rio-Celestino M Ordas A. Seed oil quality of *Brassica napus* and *Brassica rapa* germplasm from Northwestern Spain. Foods. (2019) 8:292. 10.3390/foods8080292 31357590 PMC6722933

[59] Krzyzaniak M Stolarski MJ Tworkowski J Puttick D Eynck C Załuski D. Yield and seed composition of 10 spring camelina genotypes cultivated in the temperate climate of Central Europe. Ind Crops Prod. (2019) 138:111443. 10.1016/j.indcrop.2019.06.006

[60] Cardinal AJ Burton JW Camacho-Roger AM Yang JH Wilson RF Dewey R. Molecular analysis of soybean lines with low palmitic acid content in the seed oil. Crop Sci. (2007) 47:304–10. 10.2135/cropsci2006.04.027229397403

[61] Marchler-Bauer A Derbyshire MK Gonzales NR Lu S Chitsaz F Geer LY. CDD: NCBI's conserved domain database. Nucleic Acids Res. (2015) 43:D222–6. 10.1093/nar/gku1221 25414356 PMC4383992

[62] Wilson RF Marquardt TC Novitzky WP Burton JW Wilcox JR Kinney AJ. Metabolic mechanisms associated with alleles governing the 16:0 concentration of soybean oil. J Am Oil Chem Soc. (2001) 78:335–40. 10.1007/s11746-001-0265-4

[63] Prakash NR Chhabra R Zunjare RU Muthusamy V Hossain F. Molecular characterization of *teosinte branched1* gene governing branching architecture in cultivated maize and wild relatives. 3Biotech. (2020) 10:77. 10.1007/s13205-020-2052-6 32058540 PMC6987282

[64] Gupta SK Rai AK Kanwar SS Sharma TR. Comparative analysis of zinc finger proteins involved in plant disease resistance. PLoS ONE. (2012) 7:e42578. 10.1371/journal.pone.0042578 22916136 PMC3419713

[65] Laskowski RA MacArthur MW Moss DS Thornton JM. PROCHECK - a program to check the stereochemical quality of protein structures. J Appl Cryst. (1993) 26:283–91. 10.1107/S0021889892009944

[66] Hiremath S Kumar HV Nandan M Mantesh M Shankarappa KS Venkataravanappa V. In silico docking analysis revealed the potential of phytochemicals present in *Phyllanthus amarus* and *Andrographis paniculata*, used in ayurveda medicine in inhibiting SARS-CoV-2. 3Biotech. (2021) 11:44. 10.1007/s13205-020-02578-7 33457171 PMC7799430

[67] Serrano-Vega MJ Garces R Martinez-Force E. Cloning, characterization and structural model of FatA-type thioesterase from sunflower seeds (*Helianthus annuus* L.). Planta. (2005) 221:868–80. 10.1007/s00425-005-1502-z 15841386

[68] Moreno-Perez AJ Venegas-Caleron M Vaistij FE Salas JJ Larson TR Garces R. Effect of a mutagenized acyl-ACP thioesterase *FATA* allele from sunflower with improved activity in tobacco leaves and Arabidopsis seeds. Planta. (2014) 239:667–77. 10.1007/s00425-013-2003-0 24327259

