# Supplementary material for: Allelic Variation in Zmfatb Gene Defines Variability for Fatty Acids Composition Among Diverse Maize Genotypes
Source: Front Nutr. 2022 May 6;9:845255. doi: 10.3389/fnut.2022.845255 (PMC9120846; doi:10.3389/fnut.2022.845255)
Supplement: Supplementary file 1 [file Data_Sheet_1.docx]

**Supplementary Table 1 |** Details of 48 maize inbreds used for gene-based diversity analysis using InDel-based markers

| **S. No.** | **Inbreds** | **Type** | **Source** |
| --- | --- | --- | --- |
|  | CML40 | Wild *(fatB/fatB)* | CIMMYT, Mexico |
|  | CML269 | Wild *(fatB/fatB)* | CIMMYT, Mexico |
|  | CML442 | Wild *(fatB/fatB)* | CIMMYT, Mexico |
|  | CML545 | Wild *(fatB/fatB)* | CIMMYT, Mexico |
|  | CML554 | Wild *(fatB/fatB)* | CIMMYT, Mexico |
|  | CML561 | Wild *(fatB/fatB)* | CIMMYT, Mexico |
|  | CAL1447 | Wild *(fatB/fatB)* | CIMMYT, Mexico |
|  | CML582 | Wild *(fatB/fatB)* | CIMMYT, Mexico |
|  | CAL1821 | Wild *(fatB/fatB)* | CIMMYT, Mexico |
|  | ZL17333 | Wild *(fatB/fatB)* | CIMMYT, Mexico |
|  | EC932601 | Wild *(fatB/fatB)* | USDA |
|  | EC932607 | Wild *(fatB/fatB)* | USDA |
|  | PMI-Bio-101 | Wild *(fatB/fatB)* | ICAR-IARI, New Delhi |
|  | PMI-Bio-102 | Wild *(fatB/fatB)* | ICAR-IARI, New Delhi |
|  | PMI-Bio-103 | Wild *(fatB/fatB)* | ICAR-IARI, New Delhi |
|  | PMI-Bio-104 | Wild *(fatB/fatB)* | ICAR-IARI, New Delhi |
|  | PMI-TST-103 | Wild *(fatB/fatB)* | ICAR-IARI, New Delhi |
|  | PMI-TST-105 | Wild *(fatB/fatB)* | ICAR-IARI, New Delhi |
|  | PMI-TST-106 | Wild *(fatB/fatB)* | ICAR-IARI, New Delhi |
|  | PMI-TST-107 | Wild *(fatB/fatB)* | ICAR-IARI, New Delhi |
|  | CP828-2 | Wild *(fatB/fatB)* | ICAR-IIMR, Ludhiana |
|  | CP828-1 | Wild *(fatB/fatB)* | ICAR-IIMR, Ludhiana |
|  | V335 | Wild *(fatB/fatB)* | ICAR-IIMR, Ludhiana |
|  | CM140 | Wild *(fatB/fatB)* | PAU, Ludhiana |
|  | LM5 | Wild *(fatB/fatB)* | PAU, Ludhiana |
|  | LM11 | Wild *(fatB/fatB)* | PAU, Ludhiana |
|  | BML7 | Wild *(fatB/fatB)* | PJTSAU, Telangana |
|  | CML327 | Mutant (*fatb/fatb*) | CIMMYT, Mexico |
|  | CML473 | Mutant (*fatb/fatb*) | CIMMYT, Mexico |
|  | CML563 | Mutant (*fatb/fatb*) | CIMMYT, Mexico |
|  | CML579 | Mutant (*fatb/fatb*) | CIMMYT, Mexico |
|  | CML580 | Mutant (*fatb/fatb*) | CIMMYT, Mexico |
|  | CML588 | Mutant (*fatb/fatb*) | CIMMYT, Mexico |
|  | CAL182 | Mutant (*fatb/fatb*) | CIMMYT, Mexico |
|  | CAL14137 | Mutant (*fatb/fatb*) | CIMMYT, Mexico |
|  | CAL1514 | Mutant (*fatb/fatb*) | CIMMYT, Mexico |
|  | EC932611-2 | Mutant (*fatb/fatb*) | USDA |
|  | EC932611-5 | Mutant (*fatb/fatb*) | USDA |
|  | PMI-TST-101 | Mutant (*fatb/fatb*) | ICAR-IARI, New Delhi |
|  | PMI-TST-102 | Mutant (*fatb/fatb*) | ICAR-IARI, New Delhi |
|  | PMI-TST-104 | Mutant (*fatb/fatb*) | ICAR-IARI, New Delhi |
|  | PMI-TST-108 | Mutant (*fatb/fatb*) | ICAR-IARI, New Delhi |
|  | E128 | Mutant (*fatb/fatb*) | ICAR-IIMR, Ludhiana |
|  | V409 | Mutant (*fatb/fatb*) | ICAR-VPKAS, Almora |
|  | LM13 | Mutant (*fatb/fatb*) | PAU, Ludhiana |
|  | LM17 | Mutant (*fatb/fatb*) | PAU, Ludhiana |
|  | LM25 | Mutant (*fatb/fatb*) | PAU, Ludhiana |
|  | UMI1210 | Mutant (*fatb/fatb*) | TNAU, Coimbatore |

**Supplementary Table 2 |** Details of overlapping primers developed for sequencing full length *Zmfatb* gene

| **S. No.** | **Primer name** | **Sequence (5’ 3’)** | **Length** |
| --- | --- | --- | --- |
|  | *fatb*1F  *fatb*1R | CTCCGTGTCCGCTTTTCAG  TCCCAACCGAATAAAACGAA | 19  20 |
|  | *fatb*2F  *fatb*2R | TTTTAGGTGGTGTTGTTGTGC  TTAGACAGCAAAGCGCCACT | 21  20 |
|  | *fatb*3F  *fatb*3R | CAACCATCCCATTCACACAA  CAACTGCTTCTCTGCTGCAA | 20  20 |
|  | *fatb*4F  *fatb*4R | CCCTTCCCAAGGTTAATGGT  CGTACCATGAGGGGTATTGC | 20  20 |
|  | *fatb*5F  *fatb*5R | TCACTGTGCCTCACTTTATTTTG  CATGAGAACTGTTTGTGTTGCT | 23  22 |
|  | *fatb*6F  *fatb*6R | TGGACTGATAGTTTGTGCATTTC  TAACTCCAAGGCGCATATCA | 23  20 |
|  | *fatb*7F  *fatb*7R | CCATAGCCTGCACAGTTTCA  ATGCCATTTTTCCCAGCAG | 20  19 |
|  | *fatb*8F  *fatb*8R | ATTCTGGATTCATCGGCTCA  CTGAGAGTCAGGTACAGTGGCTA | 20  23 |
|  | *fatb*9F  *fatb*9R | GACGAGCAAAGCGAGAAGTT  GATCGGAGCACTCTGTGGAC | 20  20 |
|  | *fatb*10F  *fatb*10R | TGCTAGTGCAGTGGTATCTATTGAG  CATGATGCTCAGCTCTACCAA | 25  21 |
|  | *fatb*11F  *fatb*11R | GCGACTCCACCATCCAGT  CAAGACAGAGATGGCAAGAATG | 18  22 |

F: forward, R: reverse

**Supplementary Table 3 |** Details of the primers employed for gene-based diversity in *Zmfatb* gene

| **S. No.** | **Marker** | **Sequence (5’ 3’)** | **Amplicon size (bp)** | **Region** |
| --- | --- | --- | --- | --- |
|  | *fatb*-InDel-1F  *fatb*-InDel-1R | CTCCGTGTCCGCTTTTCAG  GCCACCTCACACACAAGAGA | 84 | 5’UTR |
|  | *fatb*-InDel-2F  *fatb*-InDel-2R | CCCCCTCTCTCTCTCTCTTG  GGATTAGATACGGGCGGATT | 81 | 5’UTR |
|  | *fatb*-InDel-3F  *fatb*-InDel-3R | GGAGGCCACAAGTCCAATC  GAAAGAGCCCGGATCAAGAC | 85 | 5’UTR |
|  | *fatb*-InDel-4F  *fatb*-InDel-4R | GCCCTAATGCAGGGGATTTA  GGCACAACAACACCACCTAA | 110 | 5’UTR |
|  | *fatb*-InDel-5F  *fatb*-InDel-5R | ACTTGTCGTGTGGCCGTAGT  GGAGGACGCAAACAATTTTC | 143 | 5’UTR |
|  | *fatb*-InDel-6F  *fatb*-InDel-6R | CATCCTAAGAAATAAATGGTGTGG  TCAGTTTTTCATCCCATAAGCA | 124 | Intron 2 |
|  | *fatb*-InDel-7F  *fatb*-InDel-7R | CAGCCATAACAGACGAGCAA  CAGTGAGCCCCTTCCTAATG | 99 | Intron 3 |
|  | *fatb*-InDel-8F  *fatb*-InDel-8R | ACAACCATGCTCTGCTTCCT  GAGATTTGCATTTGGCAACA | 78 | Intron 4 |
|  | *fatb*-InDel-9F  *fatb*-InDel-9R | AGTACGAACCCGCTCCAAT  GGAGCACTCTGTGGACAAAA | 77 | Intron 5 |
|  | *fatb*-InDel-10F  *fatb*-InDel-10R | GGGGCCGACATAGTGAAG  TCCAGGTGGGAGTTTCATTC | 122 | Exon 6 |
|  | *fatb*-InDel-11F  *fatb*-InDel-11R | GAATGAAACTCCCACCTGGA  CATGATGCTCAGCTCTACCAA | 168 | 3’UTR |

F: forward, R: reverse

**Supplementary Table 4 |** List of maize genotypes and orthologue accessions of *Zmfatb* gene with their gene and protein IDs

| **S. No.** | **Accessions** | **Gene ID** | **Protein ID** |
| --- | --- | --- | --- |
|  | *Zmfatb-*Mutant1 | Nucleotide sequence generated in the present study | Protein sequence translated from nucleotide sequence |
|  | *Zmfatb-*Mutant2 |  |  |
|  | *ZmfatB-*Wild1 |  |  |
|  | *ZmfatB-*Wild2 |  |  |
|  | *ZmfatB-*Wild3 |  |  |
|  | *ZmfatB-*Wild4 |  |  |
|  | *ZmfatB-*Wild5 |  |  |
|  | *ZmfatB-*Wild6 |  |  |
|  | *ZmfatB-*Wild7 |  |  |
|  | *ZmfatB-*Wild8 |  |  |
|  | *Zea mays (Zmfatb-*B73*-*Mutant) | NC_050104.1 /  GRMZM5G829544 | NP_001357940.1 |
|  | *Brassica napus* | BnaC08g43130D | A0A078GBF8 |
|  |  | BnaA08g26890D | A0A078H139 |
|  |  | BnaAnng26510D | A0A078JN37 |
|  |  | BnaA10g09300D | A0A078GBF8 |
|  |  | BnaC05g06160D | BnaC05g06160D |
|  | *Brassica oleracea* | Bo5g009040-1 | Bo5g009040.1-1 |
|  |  | Bo5g009040-2 | Bo5g009040.1-2 |
|  |  | Bo8g112430 | Bo8g112430.1 |
|  | *Brassica rapa* | Bra018620 | Bra018620.1 |
|  |  | Bra030731 | Bra030731.1 |
|  |  | Bra031631 | Bra031631.1 |
|  | *Glycine max* | GLYMA_04G151600 | KRH63043 |
|  |  | GLYMA_05G012300 | KRH56676 |
|  |  | GLYMA_06G211300 | KRH54817 |
|  |  | GLYMA_17G120400 | KRH03792 |
|  | *Helianthus annuus* | HannXRQ_Chr05g0138201 | OTG24573 |
|  |  | HannXRQ_Chr06g0180041 | OTG23222 |
|  |  | *FATB*_HannXRQ_Chr09g0240511 | *FATB*-1_OTG13652 |
|  |  | HannXRQ_Chr10g0311291 | OTG12589 |
|  | *Citrulus lanatus* | Cla97C06G119690 | Cla97C06G119690.1 |
|  |  | Cla97C11G209120 | Cla97C11G209120.1 |
|  | *Camelina sativa* | Csa03g011960 | Csa03g011960.1 |
|  |  | Csa14g009990 | Csa14g009990.1 |
|  |  | Csa17g011970 | Csa17g011970.1 |
|  | *Chenopodium quinoa* | AUR62033409 | AUR62033409-RA |
|  | *Papaver somniferum* | C5167_012798 | RZC53943 |
|  | *Prunus dulcis* | Prudul26B003172 | VVA10652 |
|  | *P. persica* | PRUPE_7G234600 | ONH98190 |
|  | *Arabidopsis thaliana* | AT1G08510 | AT1G08510.1 |

**Supplementary Table 5** | Evolutionary distance and synonymous and non-synonymous scores in comparison with B73 reference (GRMZM5G829544) *Zmfatb* mutant allele

| **S. No.** | **Inbred** | **Pairwise distance** | **Ks** | **Ka** | **Ka/Ks** |
| --- | --- | --- | --- | --- | --- |
|  | *Zmfatb-*Mutant1 | 0.000 | 0.000 | 0.000 | 0.000 |
|  | *Zmfatb-*Mutant2 | 0.014 | 0.000 | 0.020 | 0.000 |
|  | *ZmfatB-*Wild1 | 0.023 | 0.083 | 0.007 | 0.080 |
|  | *ZmfatB-*Wild2 | 0.247 | 0.223 | 0.211 | 0.950 |
|  | *ZmfatB-*Wild3 | 0.005 | 0.020 | 0.000 | 0.000 |
|  | *ZmfatB-*Wild4 | 0.089 | 0.103 | 0.076 | 0.740 |
|  | *ZmfatB-*Wild5 | 0.038 | 0.104 | 0.020 | 0.191 |
|  | *ZmfatB-*Wild6 | 0.230 | 0.175 | 0.201 | 1.153 |
|  | *ZmfatB-*Wild7 | 0.033 | 0.061 | 0.027 | 0.439 |
|  | *ZmfatB-*Wild8 | 0.018 | 0.040 | 0.013 | 0.330 |

**Supplementary Table 6 |** *Zmfatb* gene structure parameters in maize genotypes and its selected orthologue accessions

| **S. No.** | **Accession** | **Crop** | **No. of Exons** | **Exon length (bp)** | **Exon range (bp)** | **Intron length (bp)** | **TSS (bp)** | **PolyA tail start site (bp)** | **No. of amino acids** |
| --- | --- | --- | --- | --- | --- | --- | --- | --- | --- |
|  | *Zmfatb-* B73*-*Mutant | *Zea mays* | 6 | 1305 | 69-513 | 106-1208 | 808 | 4392 | 434 |
|  | *Zmfatb-*Mutant1 | *Z. mays* | 8 | 1269 | 48-378 | 50-731 | 812 | 4402 | 422 |
|  | *Zmfatb-*Mutant2 | *Z. mays* | 6 | 924 | 36-285 | 109-1446 | 809 | 4391 | 307 |
|  | *ZmfatB-*Wild1 | *Z. mays* | 4 | 1236 | 111-513 | 104-1206 | 797 | 4369 | 411 |
|  | *ZmfatB-*Wild2 | *Z. mays* | 5 | 1275 | 132-408 | 114-1428 | 875 | 4329 | 425 |
|  | *ZmfatB-*Wild3 | *Z. mays* | 6 | 1290 | 69-513 | 105-1203 | 810 | 4376 | 429 |
|  | *ZmfatB-*Wild4 | *Z. mays* | 6 | 1002 | 111-201 | 105-1218 | 823 | 4405 | 333 |
|  | *ZmfatB-*Wild5 | *Z. mays* | 5 | 1188 | 111-480 | 111-207 | 825 | 4411 | 395 |
|  | *ZmfatB-*Wild6 | *Z. mays* | 7 | 987 | 57-420 | 104-1749 | 350 | 4350 | 328 |
|  | *ZmfatB-*Wild7 | *Z. mays* | 7 | 978 | 24-270 | 110-1291 | 838 | 4498 | 325 |
|  | *ZmfatB-*Wild8 | *Z. mays* | 7 | 1020 | 33-378 | 52-1199 | 841 | 4396 | 339 |
|  | BnaC08g43130D | *Brassica napus* | 5 | 1248 | 111-504 | 77-234 | 447 | 3296 | 415 |
|  | BnaA08g26890D | *B. napus* | 5 | 1248 | 111-507 | 77-233 | 1238 | 4172 | 415 |
|  | BnaAnng26510D | *B. napus* | 5 | 1248 | 111-504 | 79-137 | 436 | 3407 | 415 |
|  | BnaA10g09300D | *B.napus* | 5 | 1248 | 111-504 | 77-234 | 382 | 3009 | 415 |
|  | BnaC05g06160D | *B. napus* | 5 | 1239 | 111-498 | 80-86 | 385 | 3199 | 412 |
|  | Bo5g009040-1 | *Brassica oleracea* | 5 | 1239 | 111-498 | 81-88 | 385 | 2605 | 412 |
|  | Bo5g009040-2 | *B. oleracea* | 5 | 1239 | 111-498 | 86-88 | 385 | 2605 | 412 |
|  | Bo8g112430 | *B. oleracea* | 5 | 1248 | 111-504 | 88-130 | 360 | 2574 | 415 |
|  | Bra018620 | *B.rapa* | 5 | 1239 | 111-498 | 73-91 | 3 | 2593 | 412 |
|  | Bra030731 | *Brassica rapa* | 5 | 1248 | 111-507 | 80-233 | 39 | 2788 | 415 |
|  | Bra031631 | *B. rapa* | 5 | 1248 | 111-504 | 78-99 | 228 | 2699 | 415 |
|  | GLYMA_04G151600 | *Glycine max* | 6 | 1308 | 69-501 | 85-789 | 1218 | 5478 | 422 |
|  | GLYMA_05G012300 | *G. max* | 6 | 1251 | 69-492 | 107-529 | 552 | 4637 | 416 |
|  | GLYMA_06G211300 | *G. max* | 5 | 1191 | 111-492 | 371-1052 | 1009 | 5157 | 419 |
|  | GLYMA_17G120400 | *G. max* | 5 | 1125 | 111-492 | 83-625 | 551 | 4824 | 416 |
|  | HannXRQ_Chr05g0138201 | *Helianthus annuus* | 6 | 1242 | 69-498 | 90-1681 | 589 | 4146 | 413 |
|  | HannXRQ_Chr06g0180041 | *H. annuus* | 7 | 1359 | 69-501 | 84-346 | 301 | 4216 | 430 |
|  | *FATB*_HannXRQ_Chr09g0240511 | *H. annuus* | 6 | 1266 | 69-510 | 108-797 | 1931 | 6057 | 421 |
|  | HannXRQ_Chr10g0311291 | *H. annuus* | 7 | 1281 | 69-327 | 78-1589 | 705 | 5410 | 353 |
|  | Cla97C06G119690 | *Citrulus lanatus* | 8 | 1413 | 69-489 | 98-448 | 489 | 4579 | 479 |
|  | Cla97C11G209120 | *C. lanatus* | 6 | 1266 | 69-504 | 113-404 | 1554 | 4309 | 469 |
|  | Csa03g011960 | *Camellina sativa* | 5 | 1251 | 111-510 | 91-318 | 611 | 4573 | 416 |
|  | Csa14g009990 | *C. sativa* | 5 | 1251 | 111-510 | 93-225 | 489 | 3763 | 416 |
|  | Csa17g011970 | *C. sativa* | 6 | 1278 | 24-510 | 90-200 | 642 | 3992 | 416 |
|  | AUR62033409 | *Chenopodium quinoa* | 6 | 69-682 | - | - | - | - | 423 |
|  | C5167_012798 | *Papaver somniferum* | 6 | 1914 | 69-768 | - | - | - | 423 |
|  | Prudul26B003172 | *Prunus dulcis* | 6 | 1254 | 69-495 | 80-1381 | 659 | 6021 | 417 |
|  | PRUPE_7G234600 | *P. persica* | 6 | 1254 | 69-495 | 80-1382 | 575 | 5903 | 417 |
|  | AT1G08510 | *Arabidopsis thaliana* | 5 | 1239 | 111-498 | 82-231 | 741 | 4052 | 412 |

**Supplementary Table 7 |** List of domains and their features of ZmFATB protein in maize B73 reference

| **S. No.** | **Domain source** | **Start** | **End** | **Description** | **Accession** | **NCBI-CDD** |
| --- | --- | --- | --- | --- | --- | --- |
| 1. | Superfamily | 1 | 424 | Acyl-ACP-thioesterase | PLN02370 | 215210 |
| 2. | Pfam | 141 | 412 | Acyl-ACP thioesterase | pfam01643 | 366738 |
| 3. | Pfam | 1 | 130 | Acyl-thio_N, Acyl-ATP thioesterase | pfam12590 | 403703 |
| 4. | Superfamily | 141 | 411 | FatA, Acyl-ACP thioesterase | COG3884 | 226401 |
| 5. | Pfam | 150 | 260 | 4HBT | cd00586 | 238329 |
| 6. | FadM | 193 | 280 | Acyl-CoA thioesterase FadM | COG0824 | 223894 |
| 7. | Superfamily | 175 | 257 | HotDog domain superfamily | cd03440 | 239524 |
| 8. | Pfam | 319 | 408 | 4HBT_2, Thioesterase-like superfamily | pfam13279 | 404206 |

**Supplementary Table 8 |** Dock score of interaction between protein and ligands of maize and its selected orthologues of ZmFATB

| **S. No.** | **Crop** | **Accession Number** | **Similarity of protein with 5xo4** | **Dock score in binding affinity (kcal/mol)** | | | | |
| --- | --- | --- | --- | --- | --- | --- | --- | --- |
|  |  |  |  | **Palmitic acid (C_16:0_)** | **Stearic acid**  **(C_18:0_)** | **Oleic acid**  **(C_18:1_)** | **Linoleic acid**  **(C_18:2_)** | **Linolenic acid**  **(C_18:3_)** |
| 1. | Arabidopsis | AT1G08510.1 | 59.40 | -5.90 | -6.20 | -4.00 | -6.30 | -6.70 |
| 2. | Brassica napus | A0A078GBF8 | 60.15 | -6.10 | -6.40 | -6.40 | -6.10 | -6.40 |
| 3. | Brassica oleracea | Bo5g009040.1-1 | 59.77 | -6.20 | -5.80 | -6.40 | -6.30 | -6.90 |
| 4. | Brassica rapa | Bra018620.1 | 59.40 | -6.20 | -6.20 | -6.00 | -6.40 | -4.60 |
| 5. | Camelina sativa | Csa03g011960.1 | 56.76 | -6.20 | -6.10 | -6.70 | -6.20 | -6.40 |
| 6. | Citrulus sp. | Cla97C06G119690.1 | 61.98 | -6.00 | -6.10 | -6.30 | -4.70 | -6.70 |
| 7. | Maize | NP_001357940.1 | 58.73 | -6.60 | -6.50 | -6.90 | -7.3 | -6.5 |
| 8. | Opium poppy | RZC53943 | 50.38 | -5.80 | -4.30 | -4.60 | -4.80 | -5.10 |
| 9. | Prunus sp. | VVA10652 | 62.03 | -5.80 | -5.70 | -4.30 | -6.60 | -6.70 |
| 10. | Quinoa | AUR62033409-RA | 60.38 | -6.00 | -5.90 | -4.40 | -6.60 | -6.50 |
| 11. | Soybean | KRH63043 | 56.98 | -5.90 | -6.30 | -6.40 | -6.80 | -6.60 |
| 12. | Sunflower | OTG24573 | 58.71 | -5.70 | -5.90 | -6.10 | -6.40 | -6.10 |

**Supplementary Table 9 |** XYZ coordinates and number of hydrogen bonds formed during protein-ligand interaction in maize and orthologues

| **S. No.** | **Crop** | **XYZ coordinates of grid box** | **Hydrogen bonds formed during docking** | | | | |
| --- | --- | --- | --- | --- | --- | --- | --- |
|  |  |  | **Palmitic acid (C_16:0_)** | **Stearic acid**  **(C_18:0_)** | **Oleic acid**  **(C_18:1_)** | **Linoleic acid**  **(C_18:2_)** | **Linolenic acid**  **(C_18:3_)** |
| 1. | Arabidopsis  (AT1G08510.1) | X=22.0821, Y=41.8061, Z=71.5051, | VAL198, ASN320, GLU351 | VAL198, ASN320 | GLU347, ARG394 | ASN320, GLU351 | VAL198, ASN320 |
| 2. | Brassica napus  (A0A078GBF8) | X=24.3087 , Y=43.9692, Z=71.1037, | ASN322, GLU353 | VAL200, ASN322 | ASN322 | ASN322 | VAL200, ASN322 |
| 3. | Brassica oleracea  (Bo5g009040.1-1) | X=24.8163, Y=42.2066, Z=71.5859, | VAL198, ASN320, GLU351 | VAL198, GLU351 | - | ASN320, GLU351 | ASN320, GLU351 |
| 4. | Brassica rapa  (Bra018620.1) | X=21.8190, Y=42.7233, Z=72.9592, | VAL198, ASN320 | ASN320, GLU351 | VAL198, ASN320 | VAL198, ASN320 | LYS289, GLU391 |
| 5. | Camelina sativa  (Csa03g011960.1) | X=23.6053, Y=40.7414, Z=71.2539, | ASN324 | GLU355 | ASN324 | ASN324, GLU355 | VAL202, ASN324 |
| 6. | Citrulus sp.  (Cla97C06G119690.1) | X= 23.4404, Y=43.1125, Z=71.9015, | VAL240, ASN362 | VAL240, ASN362 | SER352 | - | ASN362 |
| 7. | Maize  (NP_001357940.1) | X= 25.4912, Y=41.6976, Z=73.5761, | TRP201, ASN326 | VAL203,  ASN331 | VAL203,  ASN331 | VAL203,  ASN331 | - |
| 8. | Opium poppy  (RZC53943) | X=24.4716 , Y=41.1834, Z=72.2356, | TRP196 | THR249 | - | HIS233, THR249 | GLY294 |
| 9. | Prunus sp.  (VVA10652) | X= 25.5701, Y=43.1803, Z=72.5895, | ASN319 | GLU165 | GLU390, ARG393 | SER248, TRP250, ASN319 | ASN319, GLU350 |
| 10. | Quinoa  (AUR62033409-RA) | X=23.4691, Y=42.6724, Z=73.6707, | ASN325, GLU356 | VAL203, ASN325 | GLU396, ARG399 | ASN325 | VAL203 |
| 11. | Soybean  (KRH63043) | X= 23.1357, Y=42.3764, Z=72.8496, | VAL199, ASN320 | ASN320 | ASN320 | VAL199, ASN320 | VAL199, ASN320 |
| 12. | Sunflower  (OTG24573) | X=24.0466 , Y=41.0899, Z=73.4192, | ASN320 | VAL318, ASN320 | - | VAL198, ASN320 | VAL198, ASN320 |


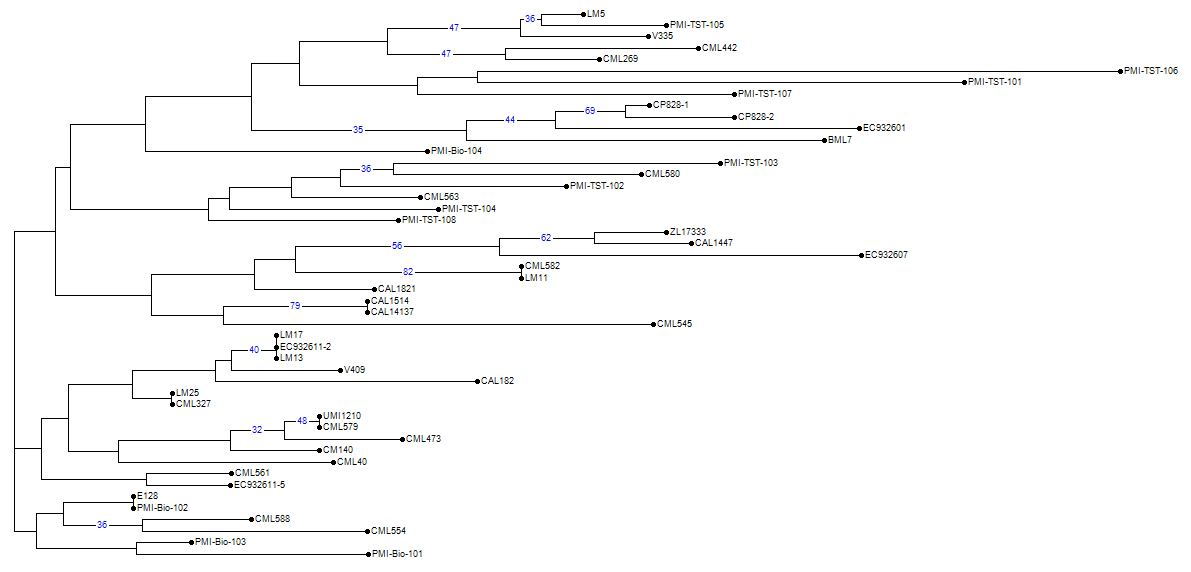


**Cluster-C1**

**Cluster-D1**

**Cluster-D2**

**Cluster-C2**

**Cluster-C**

**Cluster-D**

**Supplementary Figure 1 |** Clustering pattern of the 48 diverse inbreds using *Zmfatb* gene based InDel markers


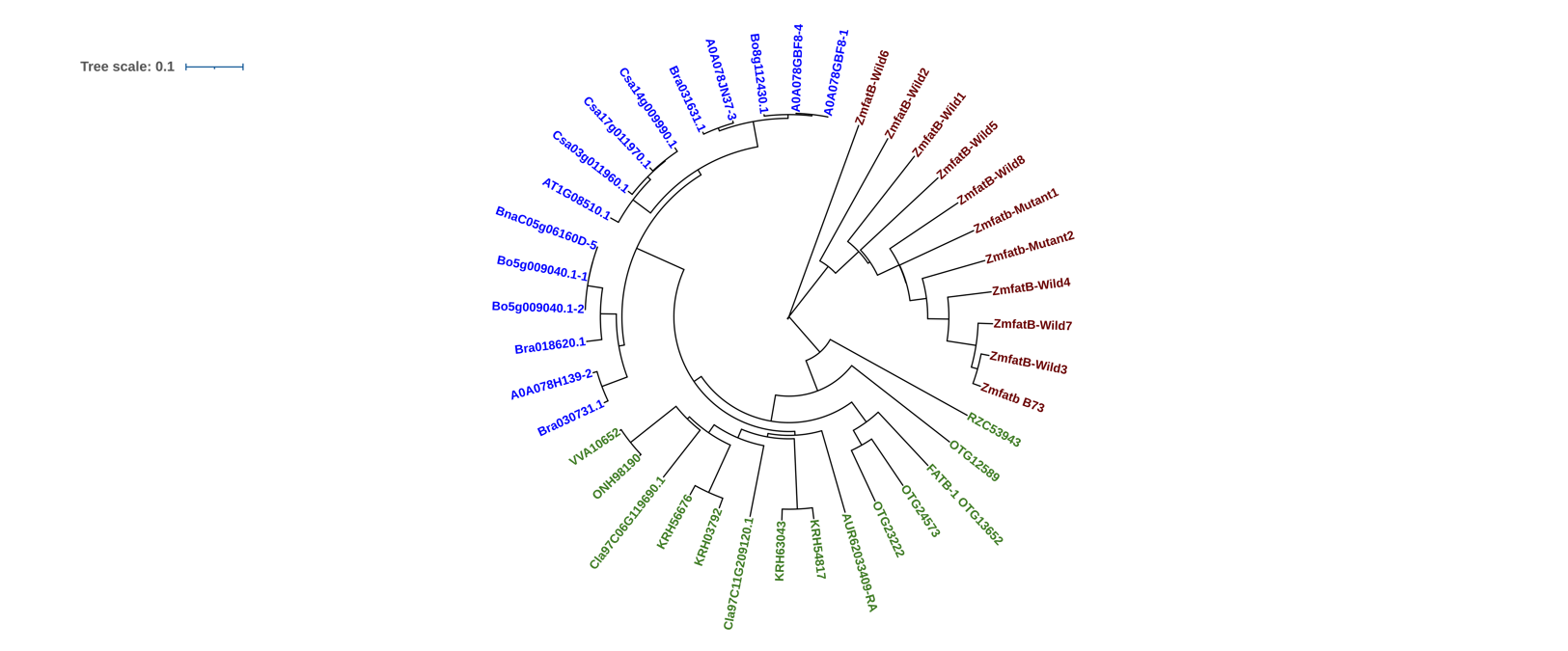


R1

R2

S


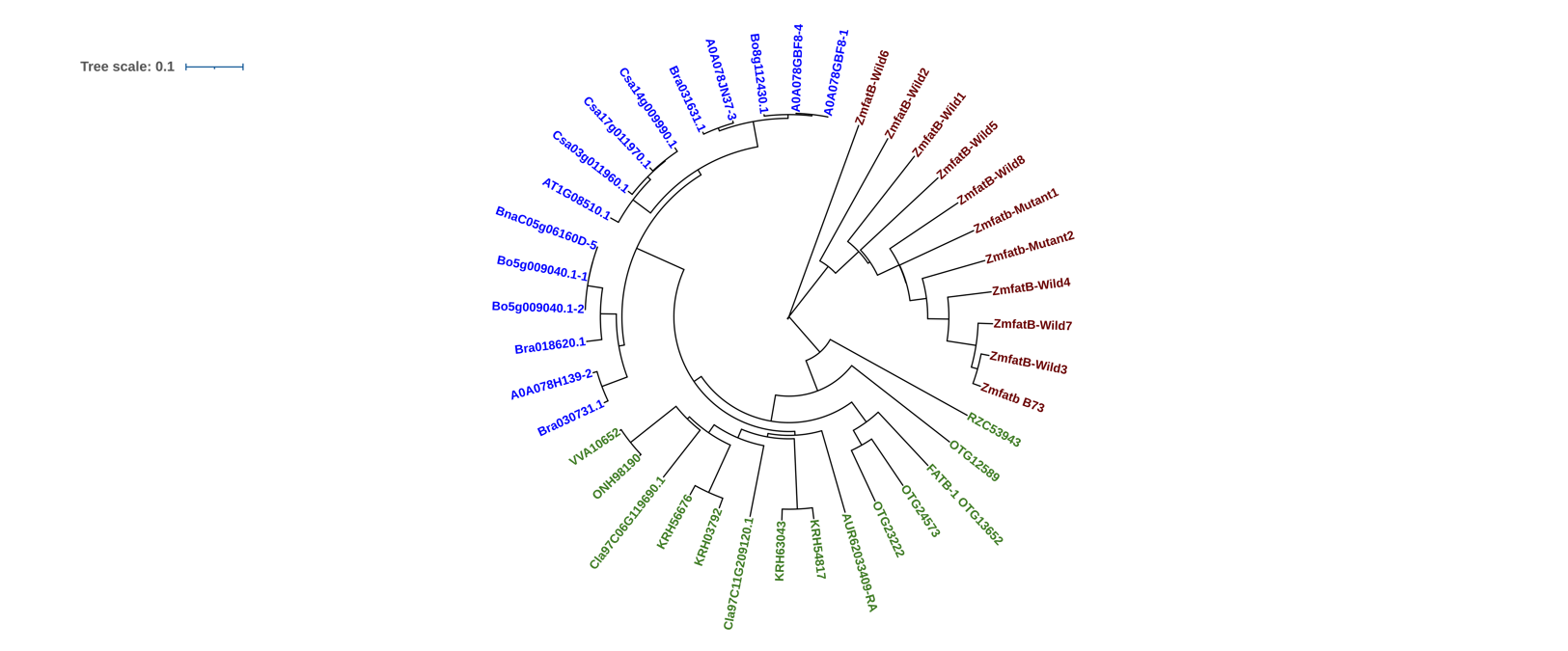


**Supplementary Figure 2 |** Protein based phylogenetic tree of maize and its orthologue accessions


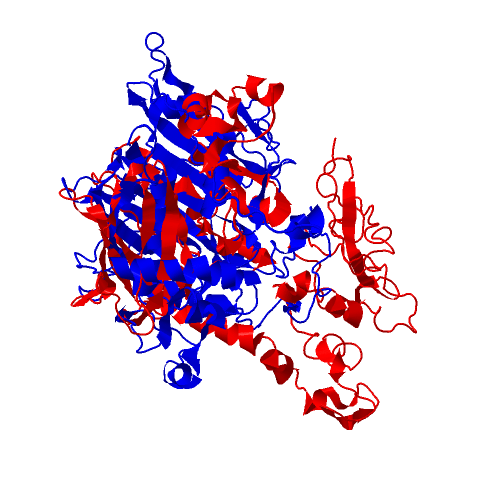


**ZmFATB-Wild1**

**ZmFATB-B73-Mutant**

**Supplementary Figure 3** | TM alignment of superimposition of mutant (ZmFATB-B73-Mutant) and wild type [ZmFATB-Wild1 (PMI-Bio-101)] proteins with TM score of 0.24196


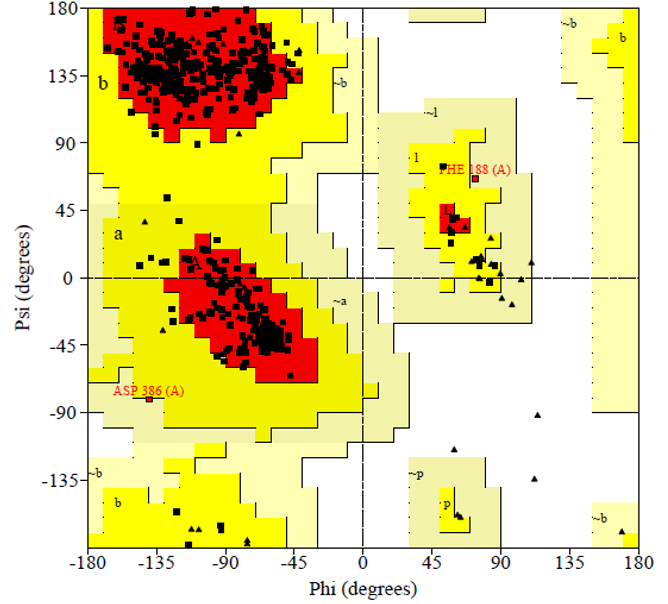


**Supplementary Figure 4a** | Ramachandran plot for mutant protein (B73 reference)


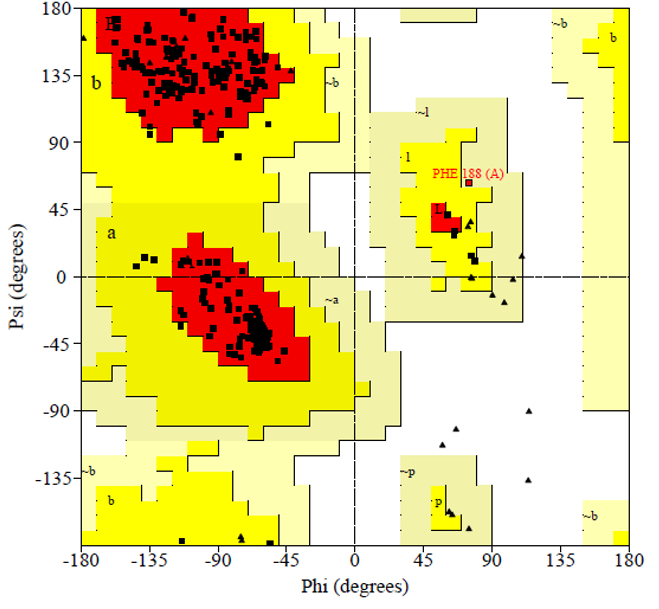


**Supplementary Figure 4b** | Ramachandran plot for wild type protein (ZmFATB-Wild1)
